# Expression and distribution of peroxiredoxins in the retina and optic nerve

**DOI:** 10.1007/s00429-015-1135-3

**Published:** 2015-10-26

**Authors:** Glyn Chidlow, John P. M. Wood, Bernard Knoops, Robert J. Casson

**Affiliations:** 1 Ophthalmic Research Laboratories, South Australian Institute of Ophthalmology, Hanson Institute Centre for Neurological Diseases, Frome Rd, Adelaide, SA 5000 Australia; 2 Department of Ophthalmology and Visual Sciences, University of Adelaide, Frome Rd, Adelaide, SA 5000 Australia; 3 Group of Animal Molecular and Cellular Biology, Institut des Sciences de la Vie (ISV), Université catholique de Louvain, 1348 Louvain-la-Neuve, Belgium

**Keywords:** Antioxidant enzyme, Immunohistochemistry, RT-PCR, Western blotting, Glia, Neurons

## Abstract

Oxidative stress is implicated in various pathological conditions of the retina and optic nerve. Peroxiredoxins (Prdxs) comprise a recently characterized family of antioxidant enzymes. To date, little information exists regarding the distribution of Prdxs in the eye. Herein, we employed a combination of qRT-PCR, immunohistochemistry and Western blotting to determine the level of expression and distribution of the six Prdx isoforms in the retina and optic nerve of the rat. In addition, we performed some parallel analyses on the common marmoset *(Callithrix Jacchus)*. In the rat, all of the Prdx transcripts were expressed in relatively high amounts in both retina and optic nerve, with abundances ranging from approximately 3–50 % of the level of the housekeeping gene cyclophilin. With regard to protein expression, each isoform was detected in the retina and optic nerve by either Western blotting and/or immunohistochemistry. Excepting Prdx4, there was a good correspondence between the rodent and primate results. In the retina, Prdx1 and Prdx2 were principally localized to neurons in the inner nuclear layer and cone photoreceptors, Prdx3 and Prdx5 displayed characteristic mitochondrial immunolabeling, while Prdx6 was associated with astrocytes and Müller cells. In the optic nerve, Prdx1 was robustly expressed by oligodendrocytes, Prdx3 and Prdx5 were observed in axons, and Prdx6 was restricted to astrocytes. The present findings augment our understanding of the distribution and expression of the Prdxs in the retina and optic nerve of rodents and primates and lay the foundation for subsequent analysis of their involvement in relevant blinding diseases.

## Introduction

Oxidative stress signifies a loss of balance between the cellular processes that produce reactive oxygen (and reactive nitrogen) species and the endogenous antioxidant defenses responsible for their elimination. Cumulative oxidative stress results in damage to DNA, proteins and lipids, and is linked to the etiology of numerous CNS disorders. The retina is particularly vulnerable to oxidative stress owing to its relatively sparse vasculature, its constant exposure to light, and most importantly its exceptional oxygen and energy demands (Ames et al. 1992). The demands of the retina are principally caused by the high metabolic rate of photoreceptors, which facilitate the processes of phototransduction and neurotransmission, but also reflect the mitochondrial-rich nature of retinal ganglion cells (RGCs), whose extensive axons remain unmyelinated prior to passing through the lamina cribrosa. Oxidative stress is hypothesized to play a key pathogenic role in development of a number of sight-threatening conditions, most notably age-related macular degeneration, diabetic retinopathy and glaucoma (Kiang et al. 2014). The ability of a tissue to withstand oxidative stress is related to the effectiveness of the endogenous antioxidant defense system, which comprises both enzymatic and non-enzymatic components.

Peroxiredoxins (Prdxs) are a family of highly conserved, low molecular weight (20–30 kD) thiol peroxidases that scavenge hydrogen peroxide, alkyl hydroperoxides, and peroxynitrite in living cells. Six Prdx isoforms (Prdx1-6) are expressed in mammalian tissues, which differ in structure, catalytic mechanisms and subcellular compartmentalization (Hanschmann et al. 2013). Prdxs1, -2 and -6 are typically located within the cytosol, Prdx3 is exclusively, and Prdx5 predominantly, localized to mitochondria, while Prdx4 is mainly reported to be associated with the endoplasmic reticulum. Interestingly, the Prdx family was not discovered until decades later than the other major cellular peroxide reducing enzymes, catalase and glutathione peroxidases (Chae et al. 1994), but owing to a combination of their high reactivity and remarkable abundance, it has been predicted that greater than 90 % of mitochondrial peroxides and 99 % of cytosolic peroxides are reduced by Prdxs (Karplus 2014). As a consequence of their status as the major cellular peroxide-removal mechanism, it is becoming apparent that Prdxs have a pivotal role to play in the response of cells to oxidative stress. Moreover, increasing evidence suggests that certain Prdxs also act as redox sensors: under conditions of oxidative stress, hyperoxidation of the Prdx molecule can occur via binding of a second peroxide substrate, resulting in the cellular buildup of Prdx oxidation products and the local accumulation of hydrogen peroxide (Rhee and Woo 2011). The physiological purpose of this reaction is not completely understood, as it necessarily entails depletion of the levels of functional Prdxs, but suggested rationalizations include the conservation of redox power during periods of oxidative stress, the initiation of downstream redox signaling pathways, and the ability of hyperoxidised Prdxs to act as protein chaperones (Karplus 2014). Hyperoxidised Prdxs have been proposed as ideal candidates to serve as endogenous biomarkers of oxidative stress (Poynton and Hampton 2014).

In order to elucidate the roles of the various Prdx isoforms in the protection of retinal neurons from oxidative stress occurring in diseases such as age-related macular degeneration, diabetic retinopathy and glaucoma, and further to investigate whether this class of antioxidant enzymes represents a suitable target for neuroprotection strategies, it is first necessary to characterize the basal distribution of the Prdx isoforms. To date, very little information is available concerning the expression of the Prdxs in the various neuronal and glial cell types of the retina and optic nerve. Prdx1 and -2 have both been identified in human retinal sections, but double labeling immunofluorescence using cell type specific markers was not performed (Klebe et al. 2014), while Prdx3 has been shown to be highly expressed in primate retina (Moreira et al. 2008). In the brain, the distribution of the Prdxs has been examined in detail (Mizusawa et al. 2000; Jin et al. 2005; Kim et al. 2008; Aon-Bertolino et al. 2011; Dammeyer and Arner 2011; Godoy et al. 2011; Goemaere and Knoops 2012). The combined data reveal that each of the isoforms displays a widespread pattern of expression, but that a marked variation exists amongst the six isoforms, both in terms of expression by specific cell types and between different brain regions.

The aim of the present study was to determine the expression and distribution of the six members of the Prdx family in the retina and optic nerve of the rat. In addition, we performed some parallel analyses on retina and optic nerve from the common marmoset *(Callithrix Jacchus)*. To achieve the goals of the study, we employed a combination of qRT-PCR, Western blotting and immunohistochemistry, using optimized primer sets and well-characterized antibodies specific to the individual Prdx isoforms.

## Materials and methods

### Animals and tissue processing

This study was approved by the SA Pathology/CHN Animal Ethics Committee (Adelaide, Australia) and conforms with the Australian Code of Practice for the Care and Use of Animals for Scientific Purposes, 2013. Adult Sprague–Dawley rats (approximately 250 g) were housed in a temperature- and humidity-controlled room with a 12-h light, 12-h dark cycle and were provided with food and water ad libitum. A total of 14 rats were used in the study. The left retinas and optic nerves of 8 rats were dissected and used for qRT-PCR. The corresponding right eyes and optic nerves were placed in Davidson’s fixative and subsequently used for immunohistochemistry. The left retinas and optic nerves of a further 6 rats were dissected and used for Western blotting. The corresponding right eyes and optic nerves were fixed in formalin. Thus, for qRT-PCR and Western blotting, each “n” represents an independent replicate. All rats were killed by transcardial perfusion with physiological saline under deep anesthesia (100 mg/kg ketamine and 10 mg/kg xylazine). Both eyes with optic nerves attached were enucleated immediately. The brain was also taken when necessary.

Human ocular tissue for analysis was obtained from the Eye-Bank of South Australia, Flinders Medical Centre (Adelaide, Australia) following the guidelines of the Southern Adelaide Clinical Human Research Ethics Committee; all had been screened to make sure there was no underlying ocular disease and all were from Caucasian donors between the ages of 50 and 65. Archived human brain tissue for analysis was obtained from the SA Brain Bank (Adelaide, Australia). Following ethical approval from the SA Pathology/CHN Animal Ethics Committee (Adelaide, Australia), we were able to obtain ocular tissue from three adult marmosets (*Callithrix jacchus*) belonging to the colony housed at the Queen Elizabeth Hospital (South Australia, Australia) that were being euthanized. We are grateful to Dr Toby Coates for participating in this tissue sharing initiative, which has obvious benefits from an ethical perspective.

Globes and optic nerves that were subsequently used for immunohistochemistry were immersion-fixed in Davidson’s solution for 24 h, then transferred to 70 % ethanol until processing. Davidson’s solution, which comprises 2 parts formaldehyde (37 %), 3 parts 100 % ethanol, 1 part glacial acetic acid and 3 parts water, is the preferred fixative for whole eyes as it provides optimal tissue morphology while avoiding retinal detachment. Alternatively, globes were immersion-fixed in 10 % buffered formalin for at least 24 h until processing. All tissues were then processed for routine paraffin-embedded sections. Globes were embedded sagittally, optic nerves longitudinally. In all cases, 4 μm sections were cut.

### Immunohistochemistry

Tissue sections were deparaffinised, rinsed in 100 % ethanol and treated for 20 min with 0.5 % H_2_O_2_ in absolute methanol to block endogenous peroxidase activity before being taken to phosphate-buffered saline (PBS). Antigen retrieval was then achieved by microwaving the sections in either 1 mM EDTA buffer, pH 8.0 (Davidson’s fixed tissue) or 10 mM citrate buffer, pH 6.0 (formalin-fixed tissue) for 10 min at 95–100 °C. Subsequently, tissue sections were blocked in PBS containing 3 % normal horse serum, incubated overnight at room temperature in primary antibody (containing 3 % normal horse serum; see Table 1), followed by consecutive incubations with biotinylated secondary antibody (1:250; Vector, Burlingame, CA) and streptavidin-peroxidase conjugate (1:1000; Pierce, Rockford, IL). Color development was achieved using NovaRed substrate kit (Vector). Sections were counterstained with haematoxylin, dehydrated, cleared in histolene and mounted in DPX.

**Table 1:** Primary antibodies

Antigen	Immunogen	Source	Dilution
PRDX1	Recombinant human peroxiredoxin 1	Hormonology Laboratory of Marloie (Belgium), rabbit polyclonal, No. UC232	1:15,000^IHC^ 1:8000^W^
PRDX2	Recombinant human peroxiredoxin 2	Hormonology Laboratory of Marloie (Belgium), rabbit polyclonal, No. UC198	1:1000^IHC^ 1:4000^W^
PRDX3	Recombinant human peroxiredoxin 3 (without mitochondrial targeting sequence)	AbFrontier; rabbit polyclonal, catalog No. LF-PA0030	1:5000^IHC^ 1:3000^W^
PRDX4	Recombinant human peroxiredoxin 4 (without secretion signal peptide)	Hormonology Laboratory of Marloie (Belgium), rabbit polyclonal, No. UC194	1:4000^IHC^ 1:3000^W^
PRDX5	Recombinant human peroxiredoxin 5 (without mitochondrial targeting sequence)	Hormonology Laboratory of Marloie (Belgium), rabbit polyclonal, No. G234	1:10,000^IHC^ 1:5000 ^W^
PRDX6	Rat recombinant non-Selenium glutathione peroxidase	Antibody Technology Australia Pty Ltd, rabbit polyclonal, catalog No. P6R	1:5000^IHC^ 1:2500^W^
β-actin	Slightly modified β cytoplasmic actin N-terminal peptide	Sigma, mouse monoclonal, clone AC-15	1:20,000^W^
Calretinin	Recombinant rat calretinin	Millipore, mouse monoclonal, clone 6B8.2	1:200^f^
Chx10	Recombinant human Chx10 protein, N-terminal	Millipore, sheep polyclonal, catalog No. AB 9016	1:1500^f^
CRALBP	Recombinant full length human CRALBP	Abcam, mouse monoclonal, clone B2	1:400^f^
Cytochrome oxidase subunit I	OxPhos IV complexes isolated from bovine heart, bovine liver, and human heart	Invitrogen, mouse monoclonal, clone 1D6	1:1000^IHC^
FGF-2	Purified bovine brain basic FGF	Millipore, mouse monoclonal, clone bFM-2	1:500^IHC^
Glutamine synthetase	Sheep glutamine synthetase aa. 1–373	BD Transduction, mouse monoclonal, catalog No. 610517	1:500^f^
Olig2	Recombinant mouse Olig-2	Millipore, rabbit polyclonal, catalog No. 9610	1:8000^IHC^
Parvalbumin	Purified frog muscle parvalbumin	Sigma, mouse monoclonal, clone PARV-19	1:400^f^
PKCα	Purified bovine brain protein kinase C	Abcam, mouse monoclonal, clone MC5	1:200^f^
SOD2	human/rat/mouse SOD2 aa. 25–43	Antibody Technology Australia Pty Ltd, rabbit polyclonal, catalog No. SOD2R	1:1000^IHC^
Vimentin	Purified vimentin from porcine eye lens	Dako, mouse monoclonal, clone V9	1:200^f^

*IHC* dilution used for 3-step colorimetric or 3-step fluorescent immunohistochemistry, *f* dilution used for 2-step fluorescent immunohistochemistry, *W* dilution used for Western blotting

For double labeling fluorescent immunohistochemistry, visualization of one antigen was achieved using a 3-step procedure (primary antibody, biotinylated secondary antibody, streptavidin-conjugated AlexaFluor 594), while the second antigen was labeled by a 2-step procedure (primary antibody, secondary antibody conjugated to AlexaFluor 488). Sections were prepared as above, then incubated overnight at room temperature in the appropriate combination of primary antibodies. On the following day, sections were incubated with the appropriate biotinylated secondary antibody (1:250) for the 3-step procedure plus the correct secondary antibody conjugated to AlexaFluor 488 (1:250; Invitrogen, Carlsbad, CA) for the 2-step procedure for 30 min, followed by streptavidin-conjugated AlexaFluor 594 (1:500; Invitrogen) for 1 h. Sections were then mounted using anti-fade mounting medium (Dako fluorescent mounting medium; Dako, Carpintería, CA) and examined under a confocal fluorescence microscope.

### Western blotting

Tissues were processed for Western blotting as previously described (Chidlow et al. 2010). In brief, entire retinas, optic nerves and brain samples were dissected and sonicated in homogenization buffer (20 mM Tris–HCl, pH 7.4, 25 °C; containing 2 mM EDTA, 0.5 mM EGTA, 1 mM dithiothreitol, 50 μg/ml leupeptin, 50 μg/ml pepstatin A, 50 μg/ml aprotinin and 0.1 mM phenylmethylsulphonyl fluoride). Note: brain tissue used as positive control tissue was taken from the cerebral cortex. An equal volume of sample buffer (62.5 mM Tris–HCl, pH 7.4, containing 4 % SDS, 10 % glycerol, 10 % β-mercaptoethanol and 0.002 % bromophenol blue) was then added and samples were boiled; protein concentrations in each sample were equalized with the bicinchoninic acid assay (Sigma-Aldrich, Sydney, NSW, Australia). Electrophoresis was performed on 12 % denaturing polyacrylamide gels after which proteins were transferred to polyvinylidene fluoride membranes (Bio-Rad, Gladesville, Australia) for immunoprobing. Membranes were incubated with the appropriate antisera (as detailed in Table 1), overnight, and labeling carried out using a multi-step detection procedure: first, appropriate biotinylated secondary antibodies were reacted with membranes and then streptavidin-peroxidase conjugates were applied. Blots were developed with a 0.016 % solution of 3-amino-9-ethylcarbazole in 50 mM sodium acetate (pH 5) containing 0.05 % Tween-20 and 0.03 % H_2_O_2_. Images were acquired from labeled blots and analyzed for densitometry using the software program, Adobe PhotoShop CS2 (Adobe Australia, Sydney, New South Wales, Australia). Densitometry values were then normalized for β-actin. Statistical comparison between the level of each isoform in retina versus optic nerve was carried out by Student’s unpaired *t* test.

### Real-time RT-PCR

Reverse transcription polymerase chain reaction (RT-PCR) studies were carried out as described previously (Chidlow et al. 2008, 2010). In brief, entire retinas and optic nerves were dissected, total RNA was isolated and first strand cDNA was synthesized from DNase-treated RNA. Real-time PCR reactions were carried out in 96-well optical reaction plates using the cDNA equivalent of 20 ng total RNA for each sample in a total volume of 20 μl containing 1× SYBR Green PCR master mix (Bio-Rad) forward and reverse primers. The thermal cycling conditions were 95 °C for 3 min and 40 cycles of amplification comprising 95 °C for 12 s, annealing temperature (Table 2) for 30 s and 72 °C for 30 s. After the final cycle of the PCR, primer specificity was checked by the dissociation (melting) curve method. In addition, specific amplification was confirmed by electrophoresis of PCR products on 3 % agarose gels. PCR assays were performed using the IQ5 icycler (Bio-Rad) and all samples were run in duplicate. Threshold cycles were calculated using IQ5 icycler Software (Bio-Rad). All values were normalized using the endogenous reference gene, cyclophilin, and results expressed as mean ± SEM.

**Table 2:** Primer sequences for mRNAs amplified by real-time RT-PCR

mRNA	Primer sequences	Product size (bp)	Annealing temperature (°C)	Accession number
GAPDH	5′-TGCACCACCAACTGCTTAGC-3′ 5′-GGCATGGACTGTGGTCATGAG-3′	87	63	NM_017008
Prdx1	5′-CCGGATGGACAATTCAAAGATA-3′ 5′-ATCCTCCTTGTTTCTTGGGTGT-3′	226	60	NM_057114
Prdx2	5′-GGCGTGTTGAAAAATGATGAG-3′ 5′-GTCCCACAGGTAGGTCGTTG-3′	103	60	NM_017169
Prdx3	5′-GGTGCTTTTCTTCTACCCTTTG-3′ 5′-CATTCTTTCTTGGCGTGTTG-3′	167	59	NM_022540
Prdx4	5′-GCAGGGCTTGGAGAGTGATG-3′ 5′-GTGCTGGCTTGGAAATCTTGG-3′	152	63	NM_053512
Prdx5	5′-AAAGGAGCAGGTTGGGAGTG-3′ 5′-GCAGATGGGTCTTGGAACAG-3′	217	63	NM_053610
Prdx6	5′-AAACTAAAACTGTCCATCCTCTACC-3′ 5′-ACCATCACACTCTCTCCCTTCT-3′	143	59	NM_053576

Primer pairs (Table 2) were designed from sequences contained in the Genbank database using the primer design software Primer 3 (http://bioinfo.ut.ee/primer3-0.4.0/primer3/) and were selected to amplify sequences that spanned at least one intron. Primer sequences were analyzed for *T*_m_ (melting temperature), secondary structure and primer-dimer formation with NetPrimer analysis software (http://www.premierbiosoft.com/netprimer) and verified both for their specificity to the target Prdx sequence and for their lack of specificity to the other Prdxs. The results showed that all six Prdx mRNAs amplified with high efficiency and linearity during real-time PCR. Mean amplification efficiencies, as determined by plotting cycle threshold as a function of initial cDNA quantity, ranged from 1.9 to 2.0. Alternative primers sets were designed and tested for each of the six Prdx mRNA species to ensure that cycle threshold values obtained were an accurate reflection of tissue abundance.

### Antibody characterization

Full details of all the antisera and antibodies used are shown in Table 1.

**Fig. 1:**
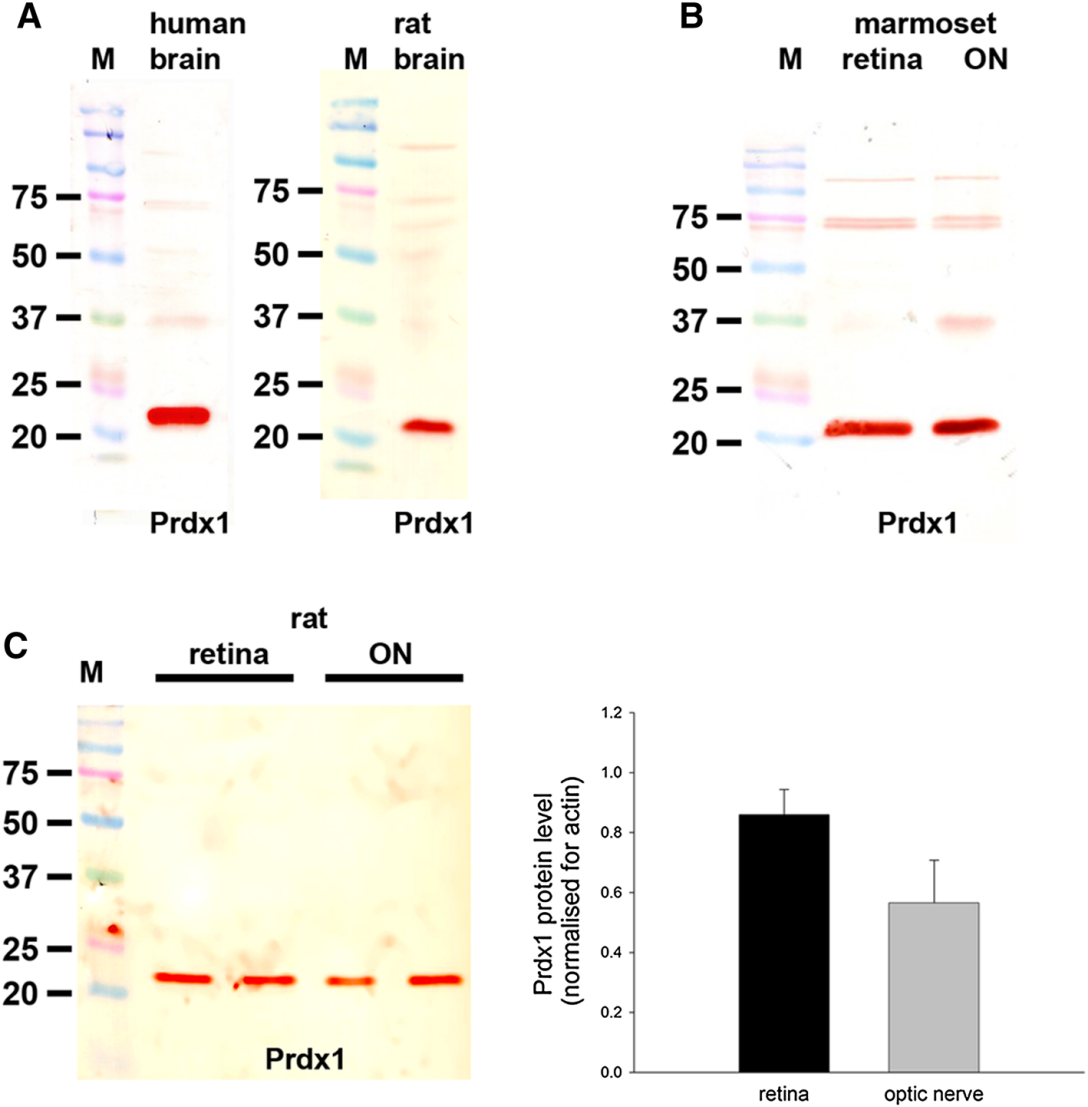
Western blot analysis of Prdx1 expression in brain, retina and optic nerve. Molecular weight markers (M, kDa) were used to determine size of detected gel products. **a** In tissue extracts from human and rat brain, a major band of the expected molecular weight (22 kDa) is apparent. **b** In tissue extracts from marmoset retina and optic nerve, a major band of the expected molecular weight is also apparent. **c** Representative immunoblots from rat retina and optic nerve tissue extracts, together with quantification of the levels of Prdx1 protein. All values (represented as mean ± SEM, *n* = 6) are normalized for actin

The polyclonal rabbit Prdx1 antiserum raised against recombinant human Prdx1 was obtained as previously described (Goemaere and Knoops 2012). It recognizes a band at 22 kDa by Western blotting performed on mouse (Goemaere and Knoops 2012), human (Fig. 1) and rat (Fig. 1) brain extracts. By immunohistochemistry, the Prdx1 antibody has been shown to produce high signal-to-background labeling in mouse brain, with robust expression observed in oligodendrocytes (Goemaere and Knoops 2012). Preadsorption with the immunizing protein eliminated Prdx1-specific staining, while incubation with preimmune serum alone gave no positive labeling (Goemaere and Knoops 2012).

The polyclonal rabbit Prdx2 antiserum raised against recombinant human Prdx2 was obtained as previously described (Goemaere and Knoops 2012). It recognizes a protein of 22 kDa by Western blotting performed on mouse (Goemaere and Knoops 2012), human (Fig. 3) and rat (Fig. 3) brain extracts. By immunohistochemistry, the Prdx2 antibody has been shown to give rise to high signal-to-background labeling in mouse brain, with an exclusively neuronal pattern of staining (Goemaere and Knoops 2012). Preadsorption with the immunizing protein eliminated Prdx2-specific staining, while incubation with preimmune serum alone elicited no labeling (Goemaere and Knoops 2012).

Rabbit anti-Prdx3 was acquired from Abfrontier (LF-PA0030). This antiserum detects a major protein band of approximately 22 kDa on western blot of mouse (Goemaere and Knoops 2012), human (Fig. 5) and rat (Fig. 5) brain extracts. A doublet band is detectable in some tissues that may correspond to a modified form of the protein (Goemaere and Knoops 2012). By immunohistochemistry, the Prdx3 antibody has been shown to be widely expressed in mouse brain neurons (Goemaere and Knoops 2012), a pattern which matched of the corresponding expression of Prdx3 mRNA (Allen Brain Atlas, Image series 70743842). Preadsorption with the immunizing protein eliminated Prdx3-specific staining.

The polyclonal rabbit Prdx4 antiserum raised against recombinant human Prdx4 was obtained as previously described (Goemaere and Knoops 2012). By Western blotting, Prdx4 antiserum recognized a 29 kDa protein in mouse brain extract with two minor additional protein bands at around 55 kDa (Goemaere and Knoops 2012). The Prdx4 staining was significantly diminished when antiserum was preadsorbed with the antigen, while incubation with preimmune serum showed a slight neuronal labeling (Goemaere and Knoops 2012). In the present study, Prdx4 antiserum revealed a single, intense band at approximately 26 kDa in human brain extracts (Fig. 8), and a less pronounced band at 26 kDa in rat brain extract with additional, higher molecular weight bands (Fig. 8). Note that mouse anti-Prdx4 from Abfontier (LF-MA0014), and four mouse anti-Prdx4 antibodies from Developmental Studies Hybridoma Bank (CPTC-PRDX4-1, CPTC-PRDX4-2, CPTC-PRDX4-3, CPTC-PRDX4-4) were tested in this study but none yielded a band at the expected molecular weight in extracts from rat retina or brain or human brain (data not shown).

The polyclonal rabbit Prdx5 antiserum raised against recombinant human Prdx5 was obtained as previously described (Wang et al. 2001). It recognizes a protein band at the expected molecular weight, 17 kDa, in human peripheral nervous tissue (Lu et al. 2006), mouse (Goemaere and Knoops 2012), human (Fig. 6) and rat (Fig. 6) brain extracts. By immunohistochemistry, the Prdx5 antibody elicited high signal-to-background labeling in mouse brain, with a prominent subcellular targeting to mitochondria (Goemaere and Knoops 2012). Preadsorption with the immunizing protein eliminated Prdx5-specific staining, while incubation with preimmune serum alone yielded no labeling (Goemaere and Knoops 2012).

Rabbit anti-Prdx6 was obtained from Antibody Technology (P6R). This antiserum detects a major protein band of approximately 26 kDa on western blot of human (Power et al. 2002) (Fig. 10) and rat (Fig. 10) brain extracts. By immunohistochemistry, the Prdx6 antibody elicited high signal-to-background labeling in human brain, with an exclusively astrocytic localization, while incubation with preimmune serum alone yielded no labeling (Power et al. 2002). This pattern of immunolabeling is identical to that produced in mouse brain using a different Prdx6 antibody (Goemaere and Knoops 2012).

For all Prdx Western blots, additional weak higher molecular weight bands seen in some tissues are related to the secondary antibody alone and typically occur when color development occurs more slowly.

Mouse anti-β-actin antibody was used in this study as a loading control in western blotting. As expected, it provided a single protein band located at 42 kDa.

Mouse anti-cytochrome oxidase subunit I (clone 1D6) was raised against OxPhos IV complexes isolated from bovine heart, bovine liver and human heart, and recognizes a single 16-kDa protein band on immunoblots (He et al. 2003). The distribution of labeling in our hands was identical to previous descriptions in retinal tissue (Mervin and Stone 2002; Raven et al. 2008).

Rabbit anti-Superoxide dismutase (SOD2) was raised against a peptide corresponding to aa. 25-43 of human/rat/mouse SOD2 (Antibody Technology, SOD2R). It recognizes a protein band at the expected molecular weight, approximately 23 kDa, by Western blotting in rat retinal and brain samples (data not shown), and demarcates a characteristic mitochondrial pattern of labeling in tissue sections.

Mouse anti-calretinin (clone 6B8.2) was used to label amacrine cells and RGCs, as has previously been shown in rat retina (Nivison-Smith et al. 2013). Specificity within the present study was confirmed by the characteristic pattern of labeling observed.

Parvalbumin is a marker of AII amacrine cells in the rat retina (Wassle et al. 1993). The specificity of clone PARV-19 for use in rat retina has been repeatedly demonstrated (Chidlow et al. 2011; Nivison-Smith et al. 2013).

The polyclonal antiserum against Chx10 protein (Millipore, ab9016) is routinely used to identify bipolar cell bodies in the retina (Chow et al. 2004; Dorval et al. 2006). The antibody reacts with a 46–50 kDa protein in rat and mouse retinal tissue (Wood et al. 2010).

Mouse anti-PKCα (clone MC5) is a widely used marker of rod bipolar cells in various species including rat (Greferath et al. 1990; Wassle et al. 1991) and marmoset (Haverkamp et al. 2003). Western blotting of rat retinal extracts reveals a single protein band of the expected molecular weight, 80 kDa (Chidlow et al. 2011).

The monoclonal antibody to FGF-2 (clone bFM-2) is highly specific for FGF-2 from bovine, human, rat, and mouse sources and does not cross-react with FGF-1. It principally recognizes a protein band at 22 kDa in rat spinal cord tissue (Tripathi and McTigue 2008). FGF-2 can be used to identify Müller cells in rat retina; it is present in a characteristic nuclear pattern of staining (Xiao et al. 1998; Chidlow et al. 2013).

Mouse anti-glutamine synthetase (clone GS-6) was used to label Müller cells, as has been previously shown in rodent retinas (Vessey et al., 2011, 2014). It recognizes a single protein band of 45 kDa on immunoblots from rat brain (see manufacturer’s data sheet) and in mouse retina (Chen and Weber 2002). The same antibody was also used to label optic nerve oligodendrocytes (Domercq et al. 1999; Chidlow et al. 2014).

CRALBP is a widely used Müller cell marker in the retina. Mouse anti-CRALBP (clone B2) recognizes a single protein band of the expected molecular weight, 36 kDa, by Western blotting of rat retina (see manufacturer’s data sheet).

The monoclonal antibody to vimentin (clone V9) has been used extensively for identifying glial cells in rat tissues. It recognizes a single protein band of the expected molecular weight, 57 kDa, when used for Western blotting (Wood et al. 2012).

The Olig-2 rabbit polyclonal antibody (Millipore, ab9610) was used in this study in order to label cells of the oligodendrocyte lineage. Olig2 transcription factor has been repeatedly shown to be expressed within oligodendrocyte nuclei (Zhou et al. 2000; Kim et al. 2011; Goemaere and Knoops 2012). Specificity within the present study was confirmed by the presence of positive nuclear labeling in a characteristic pattern in tissue sections of optic nerve and brain.

## Results

### Expression of Prdx 1-6 mRNAs in retina and optic nerve

Table 3 shows the expression levels of the mRNAs encoding Prdxs 1–6, relative to the housekeeping gene cyclophilin, in rat retina. The first point to note is that all of the Prdx transcripts are expressed in relatively high amounts in the retina with cycle thresholds varying from approximately 19–24 cycles, corresponding to 3–52 % of the abundance of cyclophilin. Secondly, comparison of the six isoforms reveals conspicuous differences in the levels of expression, with Prdx1, -2 and -5 mRNAs present in greater amounts than those encoding Prdx3, -4 and -6. Table 4 shows the expression levels of the mRNAs encoding Prdxs 1–6, relative to the housekeeping gene cyclophilin, in rat optic nerve. Overall, the Prdx transcripts are expressed at a similarly high level in the optic nerve to the retina, with cycle thresholds varying from approximately 21–25 cycles, corresponding to 3–48 % of the abundance of cyclophilin. Comparison of the six isoforms reveals Prdx1 to be expressed at the highest level in the optic nerve and Prdx3 to be least abundant. Necessarily, all of these inferences are based on the assumption that each of the primer pairs amplified with near identical efficiency. This appears to be a valid supposition, as evidenced both by preliminary analysis of plotting cycle threshold as a function of initial cDNA quantity and from analysis of alternate PCR assays using different primer sets.

**Table 3:** Levels of peroxiredoxin mRNAs in retina expressed relative to cyclophilin

	Cyclophilin (*C*_T_)	Prdx (*C*_T_)	$\Delta C_{\text{T}}^{\text{a}}$	mRNA level^b^
Prdx1	18.3 ± 0.1	20.6 ± 0.2	2.3 ± 0.1	0.22 ± 0.01
Prdx2	18.3 ± 0.1	19.3 ± 0.1	1.0 ± 0.1	0.52 ± 0.02
Prdx3	18.3 ± 0.1	22.4 ± 0.2	4.1 ± 0.1	0.07 ± 0.004
Prdx4	18.3 ± 0.1	23.6 ± 0.2	5.3 ± 0.1	0.03 ± 0.001
Prdx5	18.3 ± 0.1	20.8 ± 0.2	2.4 ± 0.1	0.20 ± 0.01
Prdx6	18.3 ± 0.1	23.1 ± 0.2	4.8 ± 0.1	0.04 ± 0.002

*C*_*T*_ cycle threshold

^a^Δ*C*_T_ Prdx *C*_T_ − cyclophilin *C*_T_

^b^Prdx mRNA level expressed relative to cyclophilin, where *n* = 8

**Table 4:** Levels of peroxiredoxin mRNAs in optic nerve expressed relative to cyclophilin

	Cyclophilin (*C*_T_)	Prdx (*C*_T_)	$\Delta C_{\text{T}}^{\text{a}}$	mRNA level^b^
Prdx1	19.5 ± 0.1	20.6 ± 0.1	1.1 ± 0.1	0.48 ± 0.02
Prdx2	19.5 ± 0.1	21.3 ± 0.1	1.9 ± 0.1	0.30 ± 0.02
Prdx3	19.5 ± 0.1	24.6 ± 0.2	5.1 ± 0.1	0.03 ± 0.003
Prdx4	19.5 ± 0.1	23.7 ± 0.1	4.3 ± 0.1	0.06 ± 0.004
Prdx5	19.5 ± 0.1	22.2 ± 0.1	2.7 ± 0.1	0.17 ± 0.02
Prdx6	19.5 ± 0.1	23.3 ± 0.2	3.8 ± 0.1	0.08 ± 0.005

*C*_*T*_ cycle threshold

^a^Δ*C*_T_ Prdx *C*_T_ − cyclophilin *C*_T_

^b^Prdx mRNA level expressed relative to cyclophilin, where *n* = 8

### Prdx1

Prdx1 is highly conserved among mammals with rodent, marmoset and human sequences displaying virtually identical homology at the protein level. The antiserum used in the current study was raised against recombinant human Prdx1 and has previously been demonstrated to recognize the murine equivalent by Western blotting and immunohistochemistry (Goemaere and Knoops 2012).

#### Western blotting

In samples prepared from human and rat brain—these tissues were used as positive controls—the Prdx1 antiserum recognized a major protein band of the expected molecular weight, 22 kDa (Fig. 1a). In tissue extracts from marmoset retina and optic nerve (Fig. 1b), as well as from rat retina and optic nerve (Fig. 1c), a band of the same molecular weight was also clearly evident. Analysis of retina and optic nerve samples from six rats revealed a quantifiable level of Prdx1 in all samples. Densitometry indicated that there was a non-significant tendency for there to be a relatively higher level of the Prdx1 protein in the retina versus the optic nerve when normalized for actin (*P* = 0.11 by Student’s unpaired *t* test; Fig. 1c).

#### Immunohistochemistry

**Fig. 2:**
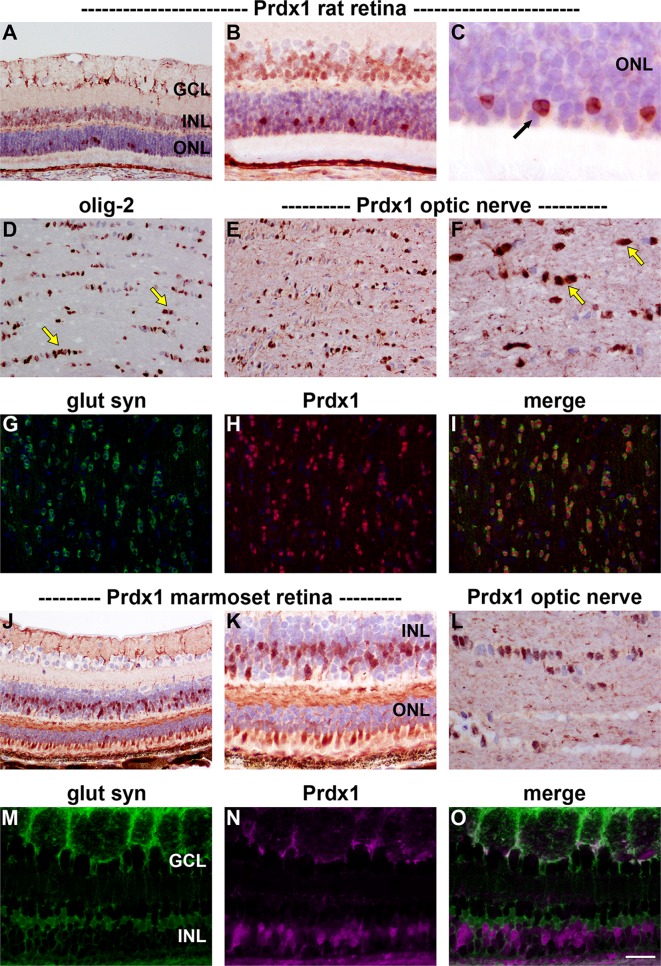
Representative images of Prdx1 immunolabeling in rat (**a**–**i**) and marmoset (**j**–**o**) retina and optic nerve. In the rat retina (**a**), positive labeling for Prdx1 is associated with processes in close proximity to retinal ganglion cells, a large population of cells in the INL—predominantly the outer INL—and with a small number of cells located within the ONL. Higher magnification images of the ONL (**b**, **c**) indicate that these intensely labeled cells (*arrow*) are cone photoreceptors owing to the presence of clumps of heterochromatin. In the optic nerve, cells with the morphology and spatial arrangement of oligodendrocytes (**d**, olig-2) are robustly labeled by the Prdx1 antiserum (**e**, **f**). In addition, weaker staining is evident within axon bundles (**e**, **f**). Double labeling immunofluorescence shows a complete co-localization of Prdx1 with glutamine synthetase in oligodendrocytes (**g**–**i**). In the marmoset, a similar pattern of distribution is observed as the rat, with prominent labeling associated with cells in the INL (**j**, **k**), with cone photoreceptors (**j**, **k**) and with optic nerve oligodendrocytes (**l**). Double labeling of Prdx1 with the Müller cell marker glutamine synthetase reveals that none of the Prdx1-positive cells within the INL are Müller cells (**m**–**o**). *Scale bar* **a**, **d**, **e**, **g**–**i**,** j** = 50 μm; **b**, **k**, *L*, **m**–**o** = 25 μm; **c** = 10 μm. *GCL* ganglion cell layer, *INL* inner nuclear layer, *ONL* outer nuclear layer

Prdx1 immunoreactivity in both rat (Fig. 2a–c) and marmoset (Fig. 2j, k) retina localized to a population of cells in the INL and to cone, but not rod, photoreceptors. Prdx1-positive somas in the INL did not express the Müller cell marker glutamine synthetase (Fig. 2m–o), indicating their status as neurons; however, Prdx1 immunolabeling was associated with some glutamine synthetase-positive processes in close proximity to retinal ganglion cells (Fig. 2m–o), suggesting that Müller cells may have limited expression of Prdx1. In rat and marmoset optic nerve, Prdx1 was robustly expressed by oligodendrocytes. In addition, weaker staining was evident within axon bundles (Fig. 2d, e, g–i, l). It is likely that oligodendrocytes are the cell type that expresses the highest amount of Prdx1 in the eye, as tenfold dilution of the anti-Prdx1 antiserum resulted in the loss of all ocular Prdx1 immunolabeling with the exception of that associated with oligodendrocytes (data not shown).

### Prdx2

Prdx2 is also highly conserved among mammals with rodent, marmoset and human sequences displaying virtually identical homology at the protein level. The antiserum used in the current study was raised against recombinant human Prdx2 and has previously been demonstrated to recognize the murine equivalent by Western blotting and immunohistochemistry (Goemaere and Knoops 2012).

#### Western blotting

**Fig. 3:**
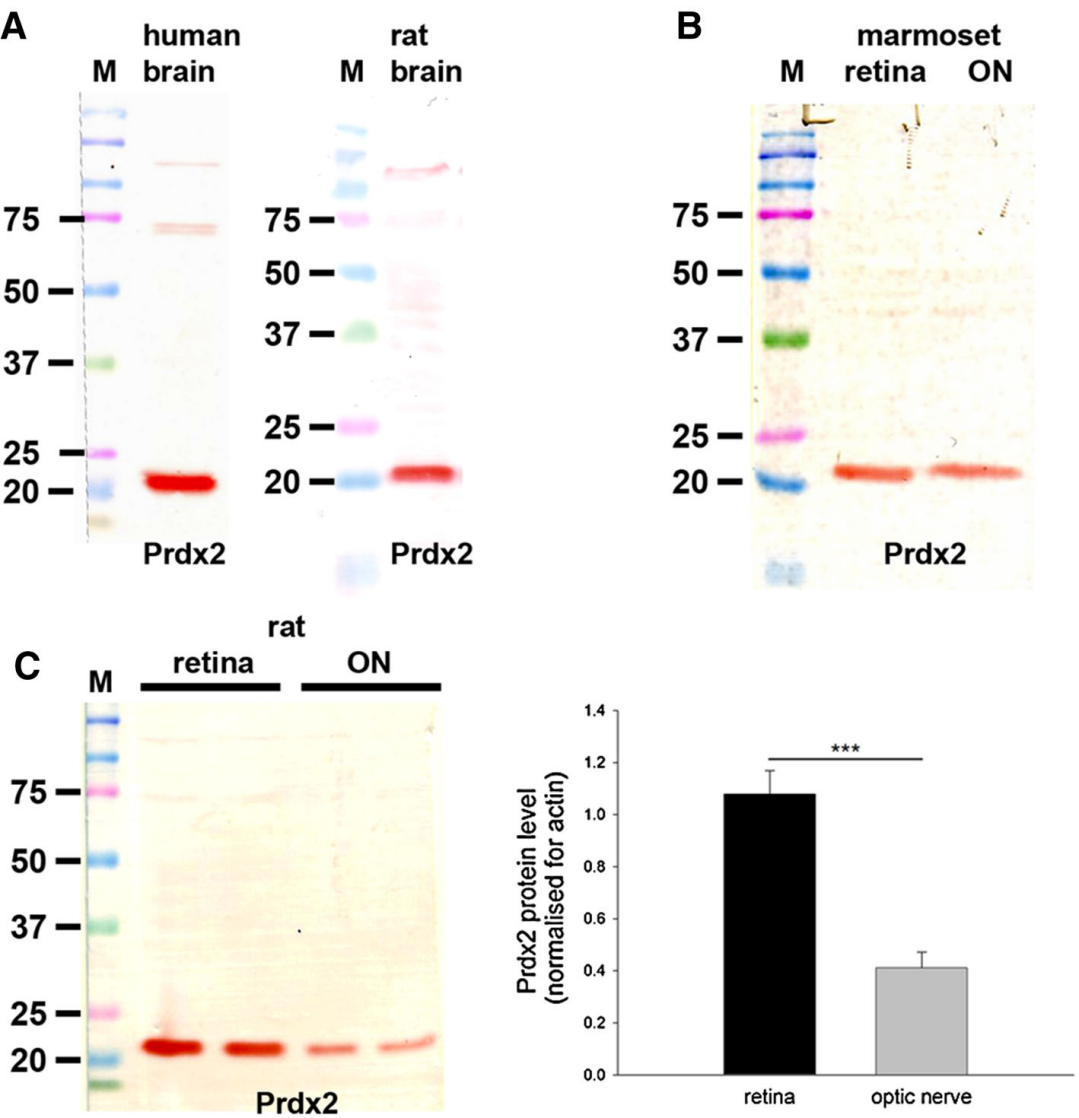
Western blot analysis of Prdx2 expression in brain, retina and optic nerve. Molecular weight markers (M, kDa) were used to determine size of detected gel products. **a** In tissue extracts from human and rat brain, a major band of the expected molecular weight (22 kDa) is apparent. **b** In tissue extracts from marmoset retina and optic nerve, a major band of the expected molecular weight is also apparent. **c** Representative immunoblots from rat retina and optic nerve tissue extracts, together with quantification of the levels of Prdx2 protein. All values (represented as mean ± SEM, *n* = 6) are normalized for actin, where ^***^ *P* < 0.001 by Student’s unpaired *t* test

In samples prepared from human and rat brain—positive control tissues—the Prdx2 antiserum recognized a major protein band of the expected molecular weight, 22 kDa (Fig. 3a). In tissue extracts from marmoset retina and optic nerve (Fig. 3b), as well as from rat retina and optic nerve (Fig. 3c), a band at the same molecular weight was also apparent. Analysis of retina and optic nerve samples from six rats revealed a quantifiable level of Prdx2 in each sample. Densitometry revealed there to be a substantially greater amount of Prdx2 in the retina versus the optic nerve when normalized for actin (*P* < 0.001 by Student’s unpaired t test; Fig. 3c).

#### Immunohistochemistry

**Fig. 4:**
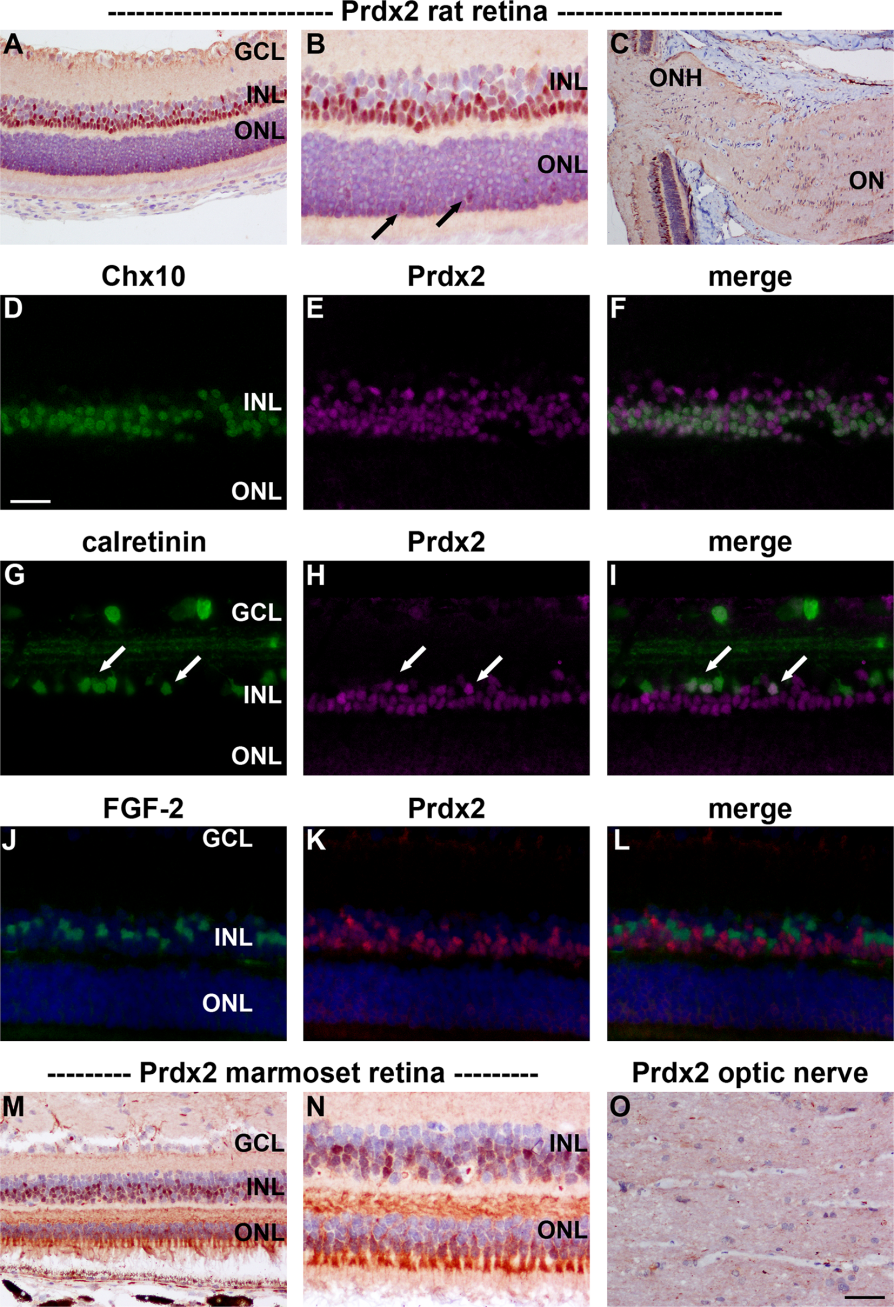
Representative images of Prdx2 immunolabeling in rat (**a**–**l**) and marmoset (**m**–**o**) retina and optic nerve. In the rat retina (**a**, **b**), positive labeling for Prdx2 is principally associated with a large population of cells in the INL, predominantly the outer INL. In addition, cone photoreceptors are weakly labeled (*arrows*). In the optic nerve, no unambiguously positive staining was associated with glial cells or axons (**c**). Double labeling immunofluorescence of the retina reveals that Prdx2 typically colocalizes with the pan bipolar cell marker Chx10 (**d**–**f**). Prdx2 is also associated with some amacrine cells, as evidenced by co-localization with calretinin (**g**–**i**). In contrast, Prdx2 is not expressed by FGF2-positive Müller cells (**j**–**l**). In the marmoset, a qualitatively similar pattern of Prdx2 distribution is observed as the rat, although expression by neurons within the INL appears slightly weaker than in the rat, while expression by cone photoreceptors is more pronounced (**m**, **n**). Marmoset optic nerve displays no obvious Prdx2 immunolabeling (**o**). *Scale bar* **c** = 100 μm; **a**, **m** = 50 μm; **b**, **d**–**l**, **n**, **o** = 25 μm. *GCL* ganglion cell layer, *INL* inner nuclear layer, *ON* optic nerve, *ONH* optic nerve head, *ONL* outer nuclear layer

Prdx2 immunoreactivity in both rat (Fig. 4a, b) and marmoset (Fig. 4m, n) retina localized to a population of cells in the INL. In addition, cone, but not rod, photoreceptors were strongly labeled in the marmoset (Fig. 4n) and weakly labeled in the rat (Fig. 4b). Double labeling experiments in the rat revealed that many Prdx2-positive cells colocalized with the pan bipolar cell marker Chx10 (Fig. 4d–f). A much smaller number of Prdx2-positive cells in the inner INL colocalized with the amacrine cell marker calretinin. In contrast, Müller cell markers, such as FGF-2 (Fig. 4j–l) and CRALBP (data not shown), labeled a discrete population of cells, indicating the absence of Prdx2 from this cell type. Surprisingly, given the high level of expression of Prdx2 mRNA and protein in rat optic nerve tissue extracts, no unambiguously positive immunostaining for Prdx2 was apparent, either in rat or marmoset optic nerve sections (Fig. 4c, o).

### Prdx3 and Prdx 5

Prdx3 and Prdx5, the mitochondrial Prdxs, are both highly conserved among mammals. The antisera used in the current study were raised against recombinant human Prdx3 and Prdx5 (both without mitochondrial targeting sequence), respectively, and have been verified as recognizing the murine equivalents by Western blotting and immunohistochemistry (Goemaere and Knoops 2012).

### Western blotting

#### Prdx3

**Fig. 5:**
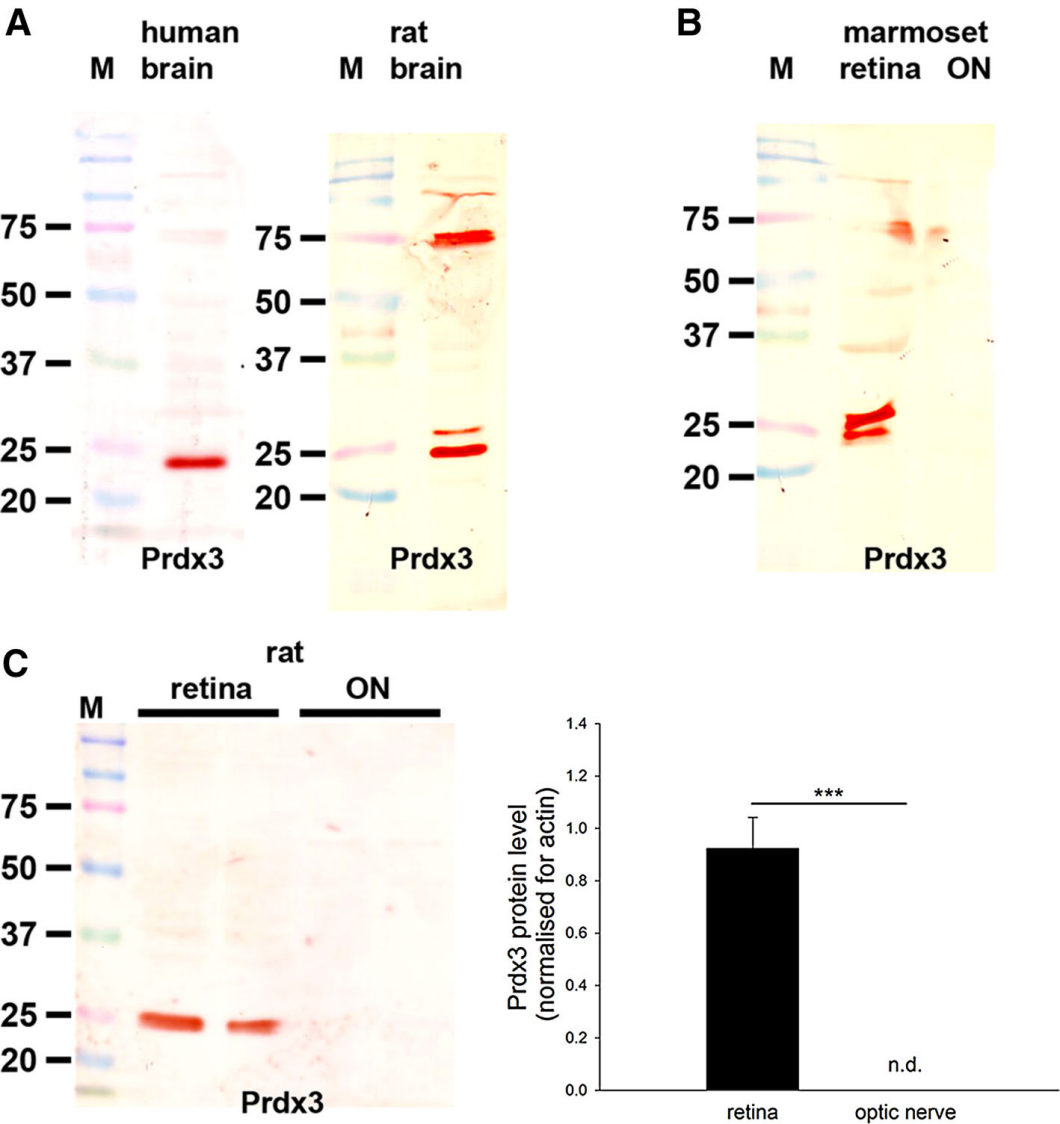
Western blot analysis of Prdx3 expression in brain, retina and optic nerve. Molecular weight markers (M, kDa) were used to determine size of detected gel products. **a** In tissue extracts from human and rat brain, a major band of the expected molecular weight (22 kDa) is apparent. In rat brain, but not retina, an additional major band is detectable at approximately 75 kD that is not thought to be related to Prdx3. **b** In tissue extracts from marmoset retina and optic nerve, a band of the expected molecular weight is also apparent. Of note, in certain samples, positive reactivity at the correct molecular weight was visualized as a doublet rather than a single band. The additional band may correspond to a modified form of the protein. **c** Representative immunoblots from rat retina and optic nerve tissue extracts, together with quantification of the levels of Prdx3 protein. All values (represented as mean ± SEM, *n* = 6) are normalized for actin, where ^***^ *P* < 0.001 by Student’s unpaired *t* test. Note: *nd* not detectable

In samples prepared from human and rat brain—positive control tissues—the Prdx3 antiserum recognized a major protein band of the expected molecular weight, 22 kDa (Fig. 5a). In tissue extracts from marmoset (Fig. 5b) and rat (Fig. 5c) retinas, positive reactivity was also apparent; however, in optic nerve samples from marmoset and rat, bands were undetectable under the assay conditions used. Analysis of retina and optic nerve samples from six rats revealed a quantifiable level of Prdx3 in each retinal extract, but not in any of the optic nerve samples (*P* < 0.001 by Student’s unpaired t test; Fig. 5c).

#### Prdx5

**Fig. 6:**
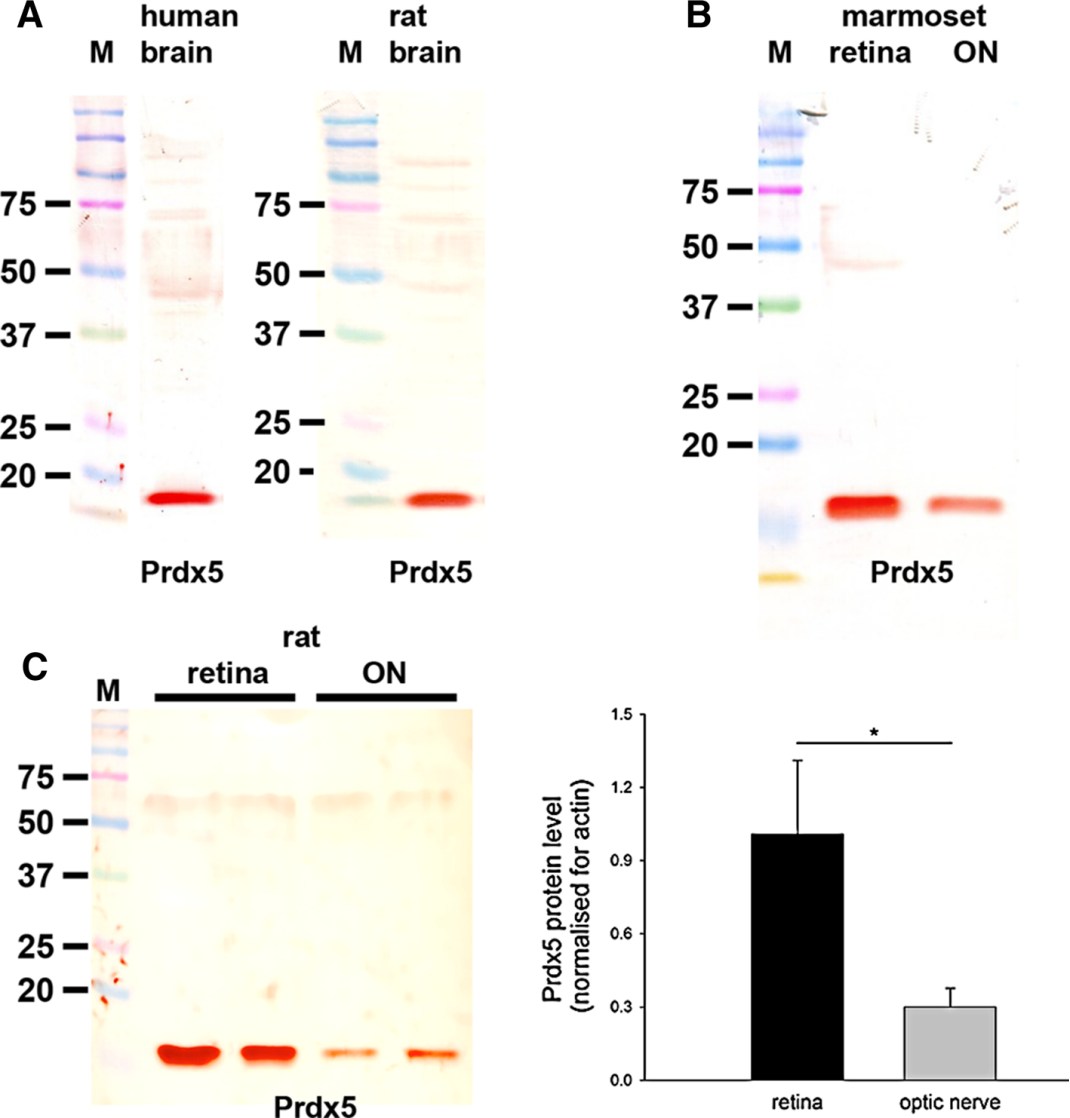
Western blot analysis of Prdx5 expression in brain, retina and optic nerve. Molecular weight markers (M, kDa) were used to determine size of detected gel products. **a** In tissue extracts from human and rat brain, a major band of the expected molecular weight (17 kDa) is apparent. **b** In tissue extracts from marmoset retina and optic nerve, a major band of the expected molecular weight is also apparent. **c** Representative immunoblots from rat retina and optic nerve tissue extracts, together with quantification of the levels of Prdx5 protein. All values (represented as mean ± SEM, *n* = 6) are normalized for actin, where ^*^ *P* < 0.05 by Student’s unpaired *t* test

In samples prepared from human and rat brain—positive control tissues—the Prdx5 antiserum recognized a major protein band of the expected molecular weight, 17 kDa (Fig. 6a) plus additional weak higher molecular weight products. In tissue extracts from marmoset retina and optic nerve (Fig. 6b), as well as from rat retina and optic nerve (Fig. 6c), a band at the same molecular weight was also apparent. Analysis of retina and optic nerve samples from six rats revealed a quantifiable level of Prdx5 in each sample. Densitometry revealed there to be a substantially greater amount of Prdx5 in the retina versus the optic nerve when normalized for actin (*P* < 0.05 by Student’s unpaired *t* test; Fig. 6c).

#### Immunohistochemistry

**Fig. 7:**
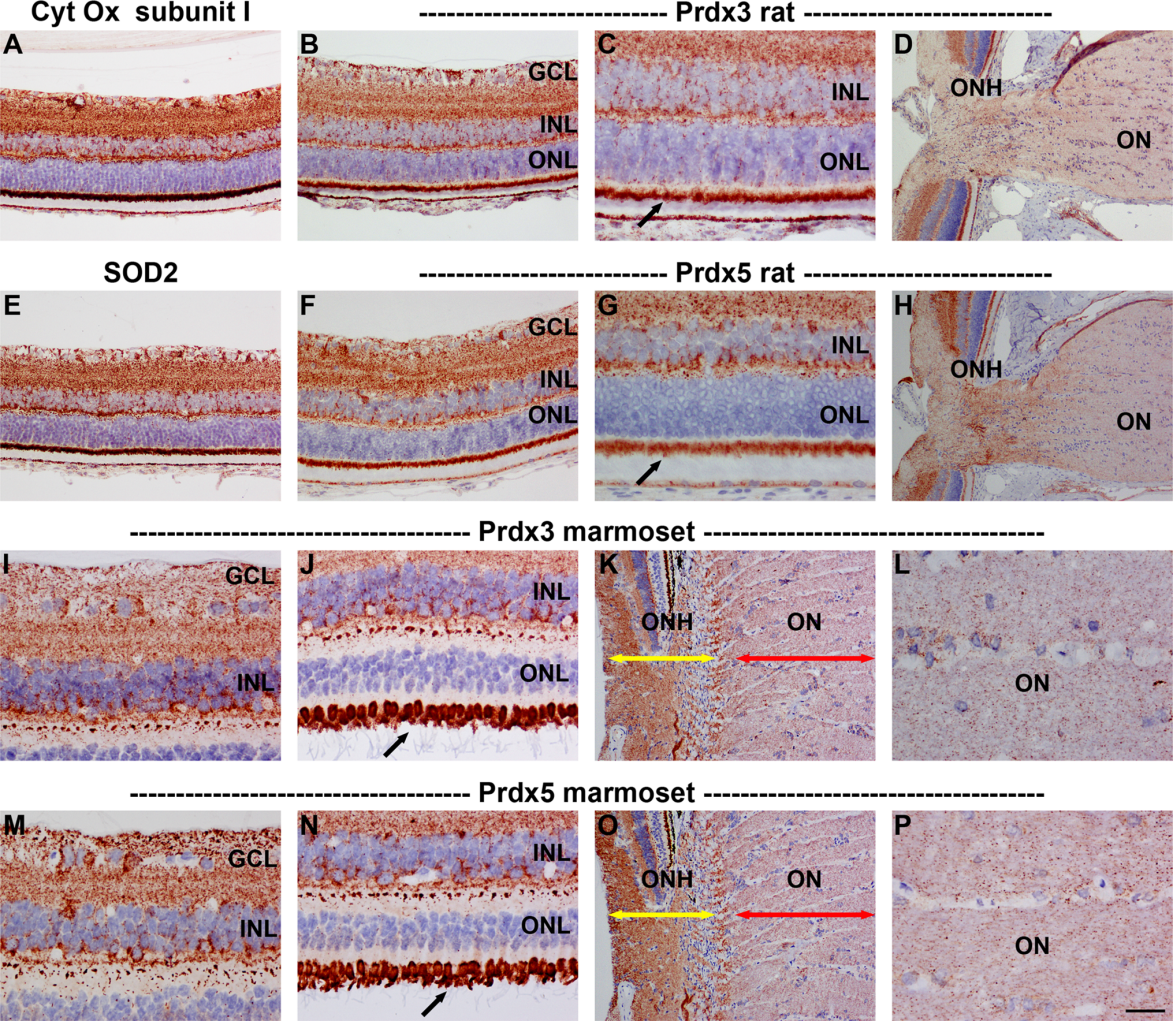
Representative images of Prdx3 and Prdx5 immunolabeling in rat (**a**–**h**) and marmoset (**i**–**p**) retina and optic nerve. Prdx3 (**b**, **c**, **i**, **j**) and Prdx5 (**f**, **g**, **m**, **n**) display very similar patterns of distribution in the rat and marmoset retina, with both antisera strongly staining the inner and outer plexiform layers and photoreceptor inner segments (*arrows*). Labeling can also be observed in the cytoplasm of various cell types, notably retinal ganglion cells. The staining patterns closely match those of other mitochondrial markers, including cytochrome oxidase (Cyt Ox) subunit I (**a**) and superoxide dismutase 2 (SOD2, **e**). Immunolabeling of sections through the ONH of rat (**d**, **h**) and marmoset (**k**, **l**, **o**, **p**) shows that both Prdx3 and 5 are present in higher abundance in the unmyelinated portion of retinal ganglion cell axons (**k**, **o**; *yellow arrows*) as compared to the zone post-myelination (**k**, **o**; *red arrows*). Higher magnification images (**l**, **p**) show that both Prdx3 and 5 are preferentially associated with axon bundles rather than glial cells in the optic nerve. *Scale bar* **d**, **h**, **k**, **o** = 100 μm; **a**, **b**, **e**, **f** = 50 μm; **c**, **g**, **i**, **j**, **m**, **n**, **l**, **p** = 25 μm. *GCL* ganglion cell layer, *INL* inner nuclear layer, *ON* optic nerve, *ONH* optic nerve head, *ONL* outer nuclear layer

Prdx3 and Prdx5 displayed patterns of distribution in the rat and marmoset retina that were strikingly similar to each other and to those of other mitochondrial markers, such as cytochrome oxidase subunit I and superoxide dismutase 2 (Fig. 7). Indeed, both Prdxs colocalized with cytochrome oxidase subunit I (data not shown). Prdx3 and Prdx5 immunoreactivities were characterized by strong, punctate labeling in the plexiform layers as well as the somas of the various cell types, notably RGCs, and also by intense labeling of photoreceptor inner segments, The superior morphology afforded by Davidson’s fixative is particularly well suited to localization of mitochondrial proteins. Interestingly, however, in sections of formalin-fixed rat retina, Prdx5 showed a greater affinity for labeling parvalbumin-positive amacrine cells than Prdx3 (data not shown).

Localization of Prdx3 and Prdx5 at the level of the optic nerve head in the rat and marmoset revealed that both proteins are present in RGC axons, with a relatively higher abundance in the proximal, mitochondrial-rich, unmyelinated portion of the optic nerve (Fig. 7d, h, k, o). Despite the apparent lack of Prdx3 in optic nerve extracts as assessed by Western blotting, both Prdx3 and Prdx5 immunoreactivities were evident in optic nerve axons (Fig. 7l, p).

### Prdx4

Prdx4 is similarly highly conserved among mammals. The antiserum used in the current study was raised against recombinant human Prdx4 (without secretion signal peptide) and has previously been demonstrated to recognize the murine equivalent by Western blotting and immunohistochemistry (Goemaere and Knoops 2012).

#### Western blotting

**Fig. 8:**
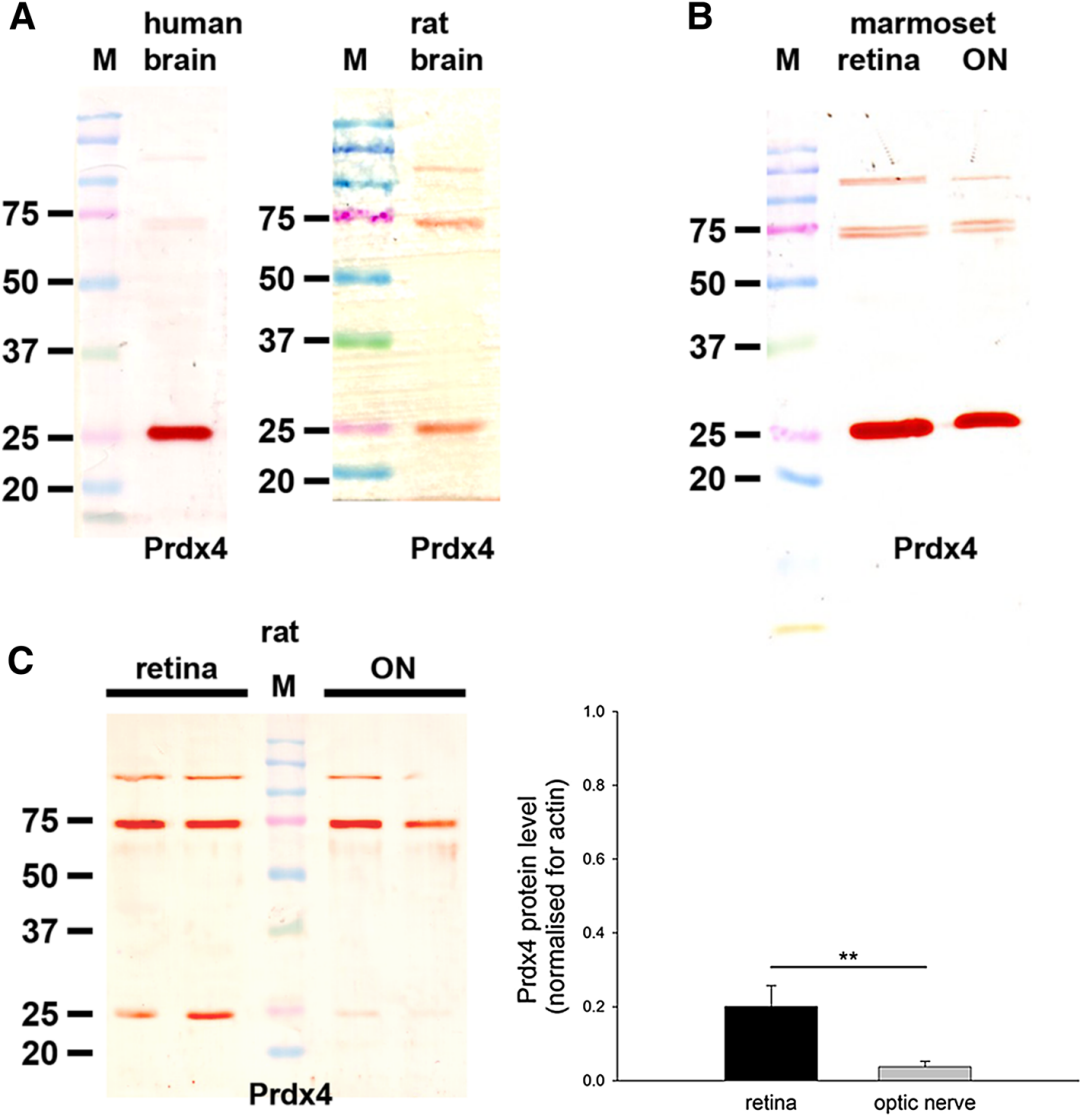
Western blot analysis of Prdx4 expression in brain, retina and optic nerve. Molecular weight markers (M, kDa) were used to determine size of detected gel products. **a** In tissue extracts from human and rat brain, a major band of the expected molecular weight (26 kDa) is apparent, which is markedly more intense in human compared with rat brain. **b** In tissue extracts from marmoset retina and optic nerve, a major band of the expected molecular weight is also apparent, which is similar to human brain in intensity. **c** Representative immunoblots from rat retina and optic nerve tissue extracts, together with quantification of the levels of Prdx4 protein. All values (represented as mean ± SEM, *n* = 6) are normalized for actin, where ^**^ *P* < 0.01 by Student’s unpaired *t* test

In the positive control tissues—human and rat brain—the Prdx4 antiserum recognized a major protein band of the expected molecular weight (26 kDa). However, the band was markedly less intense in rat brain and blots featured additional higher molecular weight bands (Fig. 8a). In marmoset retina and optic nerve extracts, an intense band of the expected molecular weight was apparent (Fig. 8b). In tissue extracts from rat retina and optic nerve (Fig. 8c), a detectable, but relatively faint, band of the correct molecular weight was evident. Densitometry revealed there to be a low amount of Prdx4 in the retina and statistically an even lower amount in the optic nerve when normalized for actin (*P* < 0.01 by Student’s unpaired *t* test; Fig. 8c).

#### Immunohistochemistry

**Fig. 9:**
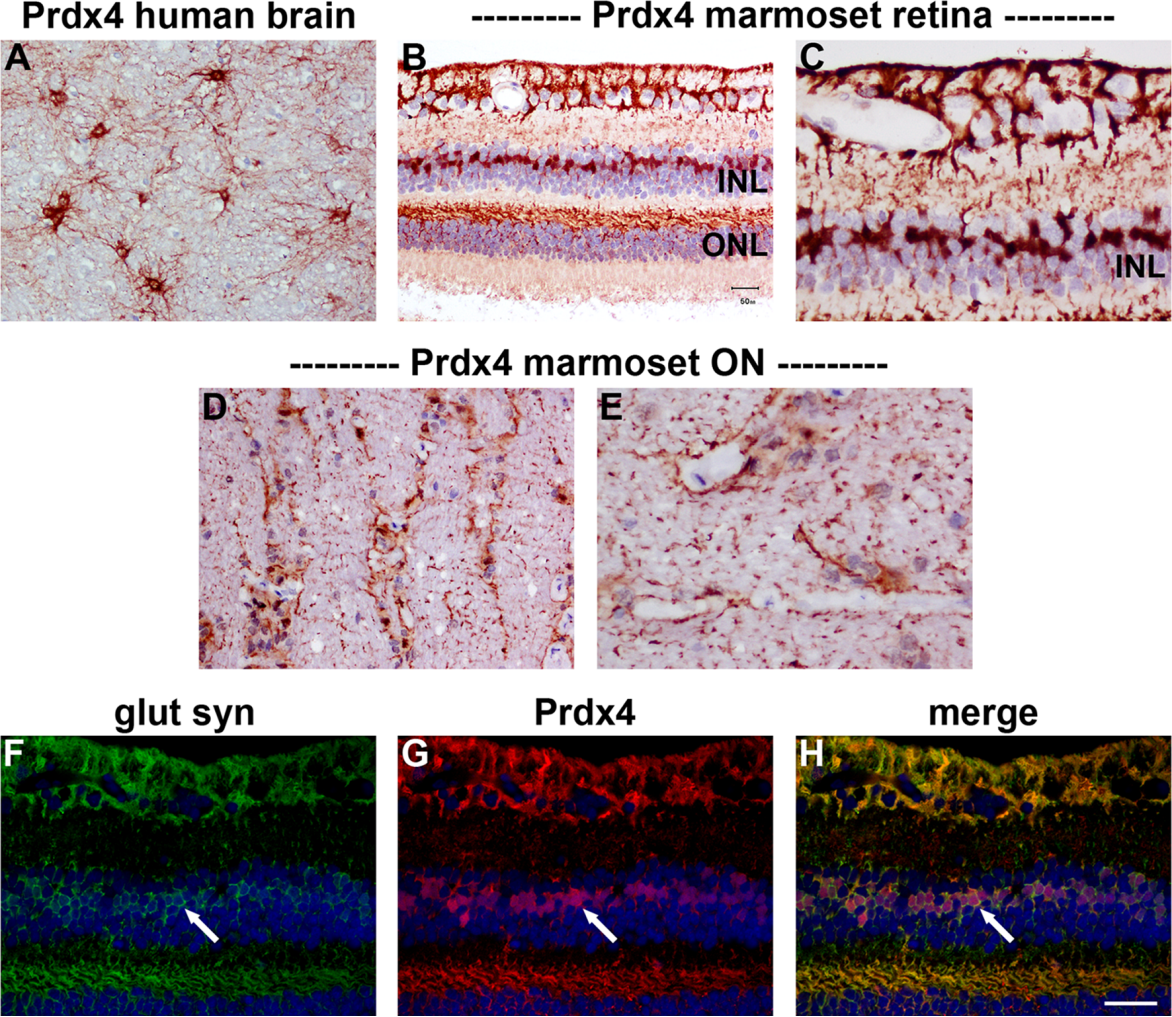
Representative images of Prdx4 immunolabeling in human brain, and marmoset retina and optic nerve. In human brain (**a**), positive labeling for Prdx4 is associated with a population of astrocytes. In marmoset retina (**b**, **c**), astrocytes and Müller cells are robustly labeled by the Prdx4 antiserum, while in the optic nerve (**d**, **e**), Prdx4 staining is distributed in a pattern characteristic of astrocytes. Double labeling immunofluorescence reveals that Prdx4 colocalizes with the Müller cell marker glutamine synthetase in the retina (glut syn; **f**–**h**, *arrows*). *Scale bar* **a**, **b**, **d** = 50 μm; **f**–**h** = 37.5 μm; **c**, **e** = 25 μm. *INL* inner nuclear layer, *ONL* outer nuclear layer

In sections of rat brain, retina and optic nerve, no unambiguously specific Prdx4 immunoreactivity was observed (data not shown). This is not unexpected for two reasons: firstly, our Prdx4 Western blotting results were unconvincing as regards rat tissue extracts; secondly, Prdx4 immunolabeling in mouse brain was shown to be minor, diffuse and not well contrasted (Goemaere and Knoops 2012). In human and marmoset tissues, however, immunohistochemical labeling with the Prdx4 antiserum produced robust, high contrast, very specific patterns of staining, which corroborated the Western blotting data. In human brain, Prdx4 was primarily associated with a population of astrocytes (Fig. 9a). In marmoset retina, strong labeling was observed in cells with the morphological characteristic of astrocytes and Müller cells (Fig. 9b, c), while in the optic nerve (D, E), Prdx4 staining was likewise distributed in a pattern characteristic of astrocytes. Confirmation of the association of Prdx4 with Müller cells was achieved by double labeling with glutamine synthetase (Fig. 9f–h).

### Prdx6

As with all of the Prdxs, Prdx6 is highly conserved among mammals. The antiserum used in the current study was raised against recombinant rat Prdx6 and has previously been demonstrated to recognize the human equivalent by Western blotting and immunohistochemistry (Power et al. 2002).

#### Western blotting

**Fig. 10:**
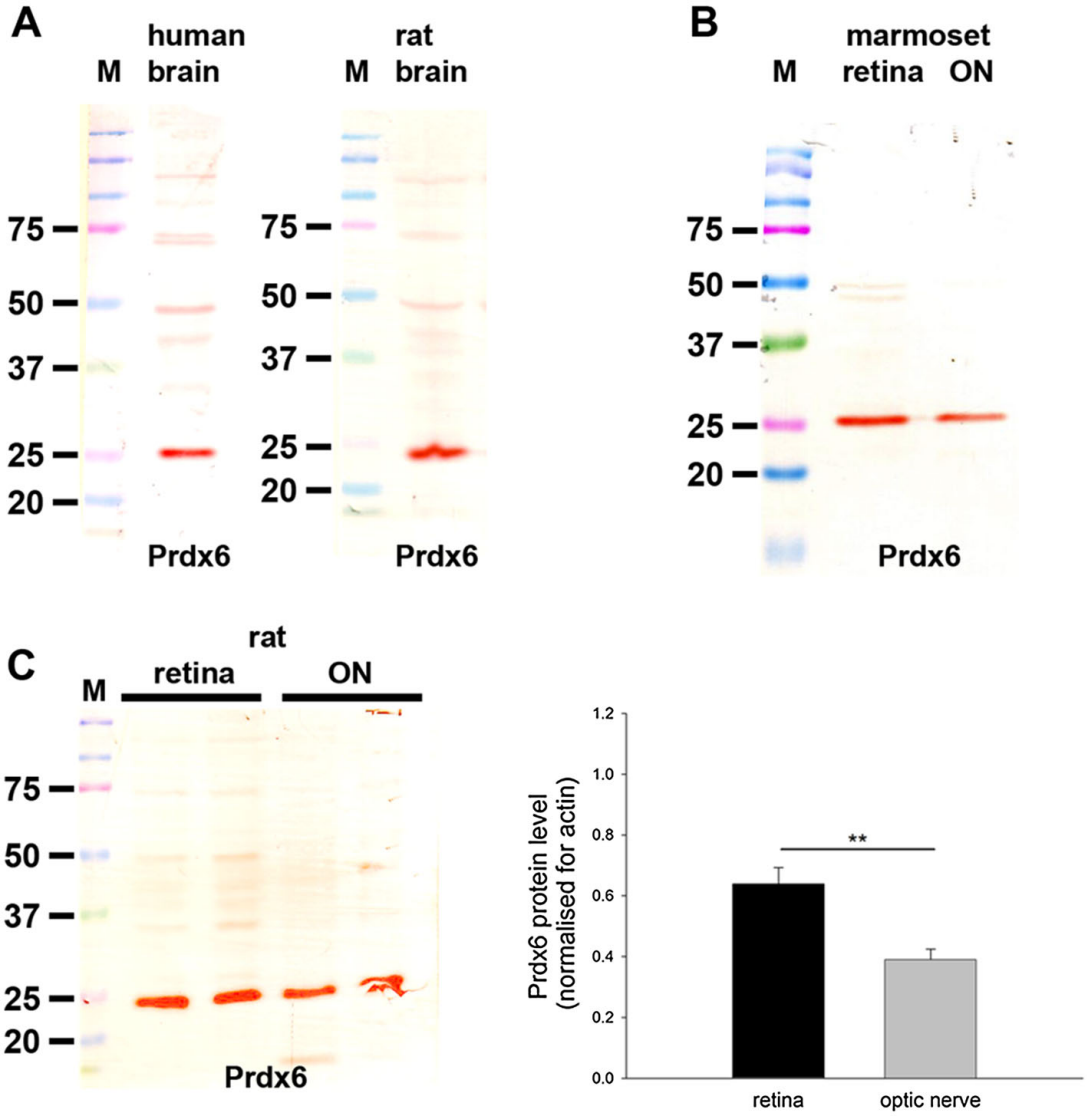
Western blot analysis of Prdx6 expression in brain, retina and optic nerve. Molecular weight markers (M, kDa) were used to determine size of detected gel products. **a** In tissue extracts from human and rat brain, a major band of the expected molecular weight (26 kDa) is apparent. **b** In tissue extracts from marmoset retina and optic nerve, a major band of the expected molecular weight is also apparent. **c** Representative immunoblots from rat retina and optic nerve tissue extracts, together with quantification of the levels of Prdx6 protein. All values (represented as mean ± SEM, *n* = 6) are normalized for actin, where ^**^ *P* < 0.01 by Student’s unpaired *t* test

In samples prepared from human and rat brain—positive control tissues—the Prdx6 antiserum recognized a major protein band of the expected molecular weight, 26 kDa (Fig. 10a). In tissue extracts from marmoset retina and optic nerve (Fig. 10b), as well as from rat retina and optic nerve (Fig. 10c), a band at the same molecular weight was also evident. Analysis of retina and optic nerve samples from six rats revealed a quantifiable level of Prdx6 in each sample. Densitometry indicated that there was a higher level of the Prdx6 protein in the retina versus the optic nerve when normalized for actin (*P* < 0.01 by Student’s unpaired *t* test; Fig. 10c).

#### Immunohistochemistry

**Fig. 11:**
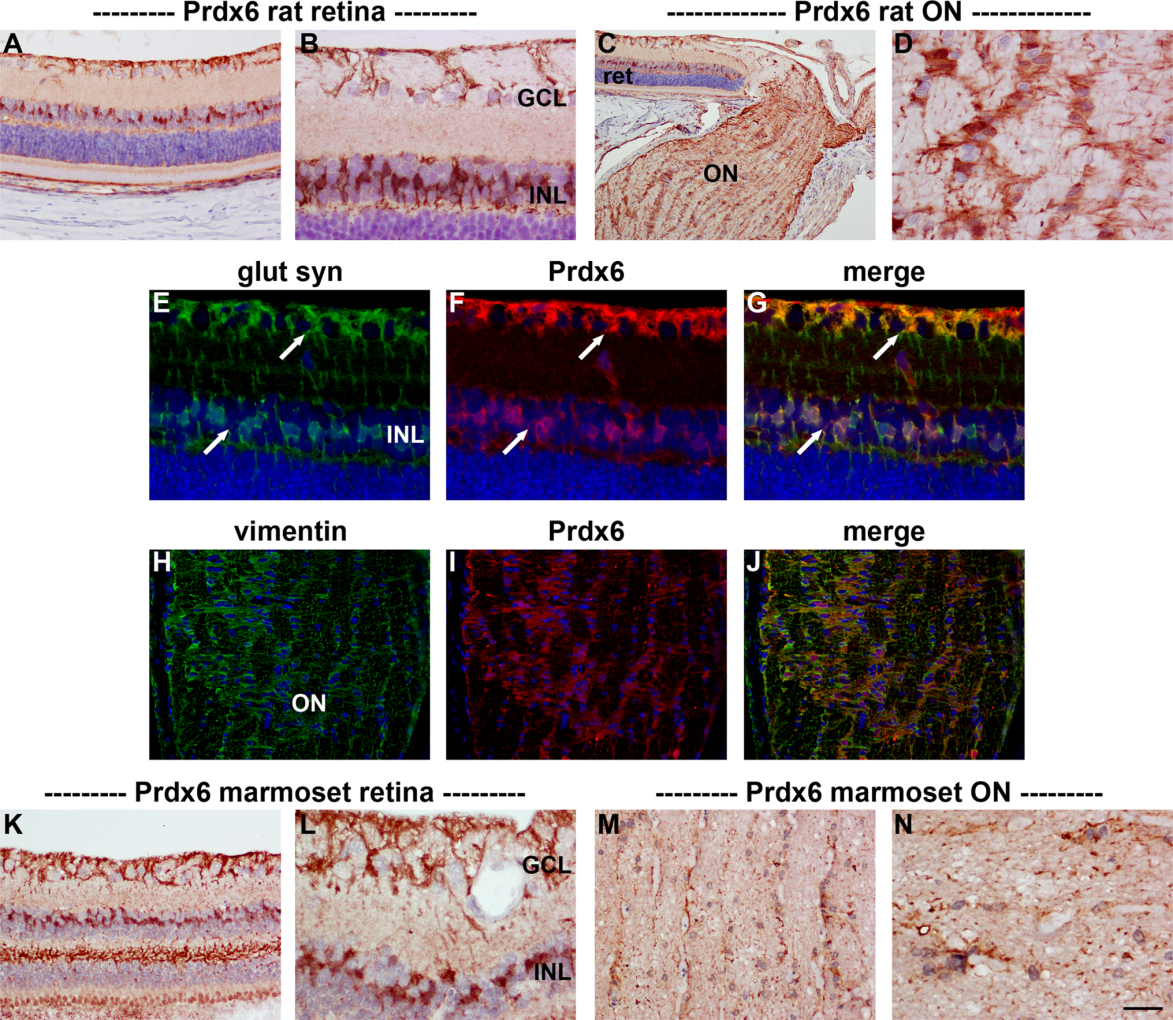
Representative images of Prdx6 immunolabeling in rat (**a**–**j**) and marmoset (**k**–**n**) retina and optic nerve. In the rat retina (**a**, **b**), positive labeling for Prdx6 is associated with a population of cells in the INL, with putative Müller cell end feet, and with astrocytes in the nerve fibre layer. In the optic nerve, Prdx6 staining is distributed in a pattern characteristic of astrocytes (**c**, **d**). Double labeling immunofluorescence of the retina reveals that Prdx6 colocalizes with the Müller cell marker glutamine synthetase (glut syn; **e**–**g**, *arrows*). In the optic nerve, Prdx6 colocalizes with the astrocytic marker vimentin (**h**–**j**). In the marmoset, a similar pattern of Prdx6 distribution in the retina (**k**, **l**) and optic nerve (**m**, **n**) is observed as the rat, although expression by optic nerve astrocytes is typically weaker than in the rat. Note the robust labeling of Prdx6 in Müller cell processes within the Henle fiber layer (**k**). *Scale bar* *C* = 100 μm; **a**, **h**–**j**, **k**, **m** = 50 μm; **b**, **d**, **e**–**g**, **l**, **n** = 25 μm. *GCL* ganglion cell layer, *INL* inner nuclear layer, *ON* optic nerve, *ret* retina

In sections of rat brain, Prdx6 was associated with GFAP-positive astrocytes (data not shown). In the eye, Prdx6 was expressed by cells with the morphological characteristics of Müller cells and astrocytes in the retina (Fig. 11a, b) and with astrocytes in the optic nerve (Fig. 11c, d). Prdx6 was observed to colocalize with the Müller cell markers glutamine synthetase (Fig. 11e–g) and FGF-2 (data not shown) in the retina and with the astrocytic marker vimentin in the optic nerve (Fig. 11h–j). There was no evidence of any association of Prdx6 with retinal neurons, RGC axons or oligodendrocytes. A similar pattern of Prdx6 distribution was seen in the marmoset retina (Fig. 11k, l) and optic nerve (Fig. 11m, n), although expression by optic nerve astrocytes appeared less robust than in the rat and less pronounced than was observed for Prdx4.

## Discussion

The retina is continually exposed to a high level of free radicals owing to its extremely high metabolic rate combined with the biooxidation effect of light. Accordingly, endogenous antioxidants, such as Prdxs, play crucial roles in protecting vulnerable retinal neurons from oxidative stress (Sacca et al. 2013). In order to begin to elucidate whether the various Prdx isoforms are potential targets for therapy during diseases such as age-related macular degeneration, diabetic retinopathy and glaucomatous optic neuropathy, we have characterized the basal levels of expression and cellular distributions of the six isoforms of the Prdx family in the retina and optic nerve of rodents and primates. In the retina, Prdx1 and Prdx2 were principally localized to neurons in the inner nuclear layer as well as cone photoreceptors, Prdx3 and Prdx5 displayed characteristic mitochondrial patterns of immunolabeling, while Prdx6 was associated with astrocytes and Müller cells. In the optic nerve, Prdx1 was robustly expressed by oligodendrocytes, Prdx3 and Prdx5 were observed in axons, and Prdx6 was restricted to astrocytes. Prdx2, while present in Western blotting in optic nerve extracts, was not readily detectable via immunohistochemistry.

### Technical considerations

Preliminary, high throughput screening of the distribution profiles of the Prdx isoforms in the rat retina has previously been conducted within the context of a wide-ranging study that examined expression of the thioredoxin, glutaredoxin and Prdx families throughout the CNS (Aon-Bertolino et al. 2011). Our findings display elements of commonality with the results of this study, most notably with regard to the pattern of Prdx3 localization; however, detailed comparison is challenging owing to the fact that the authors of the earlier work were unable to discriminate between glial and neuronal labeling in tissue sections and did not perform any Western blotting using retinal extracts. Methodological differences inevitably account for the vast majority of non-correspondence between analogous studies, but since categorical demarcation of specific and non-specific binding is problematic, caution is advisable when ascribing a pattern of immunolabeling as definitive. Indeed, significant disparity is evident between the results of different studies that have examined the distribution of Prdxs in the brain (Mizusawa et al. 2000; Jin et al. 2005; Kim et al. 2008; Aon-Bertolino et al. 2011; Dammeyer and Arner 2011; Godoy et al. 2011; Goemaere and Knoops 2012). With regard to the present study, the Prdx1-5 antibodies have been extensively validated in our previously published work in rodent brain (Goemaere and Knoops 2012), while the Prdx6 antibody has been carefully verified for use in human brain tissue (Power et al. 2002, 2008).

Quantitative comparison of the different Prdx isoforms in rat retina and optic nerve was conducted by qRT-PCR and to a limited extent by Western blotting. The latter technique is not reliably quantifiable when used for the purposes of comparing the endogenous levels of multiple proteins, for example the Prdxs, in a given tissue, owing to the differing and unknown affinities of each antiserum for its target protein. Quantitative inferences can, however, be tentatively drawn with regard to relative endogenous expression of a known protein, for example Prdx1, in different tissues, as the affinity of the antibody should become a constant factor. Given the quantitative limitations of Western blotting, the use of qRT-PCR to measure the mRNA levels of each Prdx isoform was a worthwhile inclusion in the present study. The advantages of qRT-PCR relate to its specificity, sensitivity and linearity, which yield data that are quantitatively much more reliable. Inclusion of mRNA data imparts valuable perspective on the protein results.

### Prdx1 and Prdx2

In the optic nerve, Prdx1 was expressed principally by oligodendrocytes. The finding is in good agreement with the results of various studies in the brain, all of which demonstrate that Prdx1 localizes to oligodendrocytes (Mizusawa et al. 2000; Jin et al. 2005; Kim et al. 2008; Goemaere and Knoops 2012). We also delineated a weak association of Prdx1 with axons, an observation previously noted in cerebral white matter (Mizusawa et al. 2000). With regard to Prdx2, both the qRT-PCR and Western blotting data showed there to be a clearly detectable amount of this isoform, yet, the immunohistochemistry results were inconclusive. There was no unambiguous localization of Prdx2 to glial cells in the optic nerve, a finding predicted by the lack of Prdx2 expression in brain glia (Jin et al. 2005; Hattori and Oikawa 2007; Goemaere and Knoops 2012). The logical inference, therefore, is that Prdx2 is present within RGC axons. This possibility cannot be discounted from scrutiny of immunolabeled tissue sections; nevertheless, it might be anticipated that there would be an accompanying strong association of Prdx2 with RGC somas, as was the case for Prdx3 and Prdx5. This was not evident. In order to resolve the issue, experiments are needed using rats subjected to optic nerve transection, which causes the loss of RGCs and Wallerian degeneration of their axons without accompanying death of glial cells.

In the retina, Prdx1 protein was localized to a population of cells in the inner nuclear layer and also to cone photoreceptors. A broadly similar pattern of Prdx1 immunolabeling has recently been observed in human retinal sections (Klebe et al. 2014). The Prdx1-positive cells in the inner nuclear layer were uniformly negative for Müller cell markers indicating their status as neurons, which, coupled with rudimentary inspection of their location, putatively identifies them as bipolar cells. As the marmoset retina contains many subclasses of bipolar cells (Chan et al. 2001; Weltzien et al. 2014), further investigation is needed to identify which specific types express Prdx1. In the brain, Prdx1 has been shown either not to be expressed by NeuN-positive neurons (Jin et al. 2005; Kim et al. 2008), or only very weakly (Goemaere and Knoops 2012). This was also true of the NeuN-expressing neurons of the retina, namely RGCs and amacrine cells. Bipolar cells and photoreceptors, however, are both highly specialized neurons that do not express quintessential neuronal markers such as NeuN (Wolf et al. 1996) and PGP 9.5 (Wilson et al. 1988). Finally, while we did not detect Prdx1 in Müller cell somas, co-localization of signal was evident in some glutamine synthetase-positive Müller cell endfeet. By way of reference, Prdx1 is weakly associated with astrocytes in the brain (Sarafian et al. 1999), and in particular with perivascular astrocytic feet (Mizusawa et al. 2000; Goemaere and Knoops 2012). Prdx2 was localized exclusively to neurons, specifically to Chx10-positive bipolar cells, to some calretinin-expressing amacrine cells, and also to cone photoreceptors. A comparable pattern of Prdx2 immunolabeling has recently been reported in human retina (Klebe et al. 2014). In many respects, the localization pattern of Prdx2 resembled that of Prdx1, however, some differences were evident: in the rat, Prdx2 labeling was more robust than Prdx1 in bipolar cells, less intense than Prdx1 in cone photoreceptors, and absent from putative Müller cell end feet. Conversely, in the marmoset, Prdx2 was less robust than Prdx1 in inner retinal neurons, but more intense than Prdx1 in cone photoreceptors, and likewise absent from putative Müller cell end feet. The exclusively neuronal localization of Prdx2 matches the reported cellular distribution of this isoform in brain tissue (Jin et al. 2005; Hattori and Oikawa 2007; Goemaere and Knoops 2012). In the brain, as in the retina, there is marked variation in Prdx2 expression between different neuronal populations.

#### Implications for disease

Of the retinal neuronal classes, bipolar cells are remarkably resilient to degeneration in animal models of injury. Our study shows that bipolar cells, uniquely, express both Prdx1 and -2 isoforms, a finding that may help explain their resistance to oxidative injury. With regard to photoreceptors, rod photoreceptors displayed negligible expression of Prdx1 and -2, but cones were associated with both cytosolic Prdx isoforms. Prdx2 hyperoxidation has been detected in extracts from a pro-oxidant-treated cultured photoreceptor cell line (Rezaie et al. 2012), signifying the role of this protein in buffering oxidative stress. It can be hypothesized that the absence of Prdx1 and -2 renders rods more vulnerable than cones to oxidative stress, and may help explain why rod loss is greater than cone loss during aging and in both the early and late stages of age-related macular degeneration (Curcio et al. 2000). The use of Prdx1 and -2 knockout and over-expressing mice would help test this hypothesis. Of note, in the brain, transgenic mice overexpressing Prdx2 displayed reduced injury and improved neurological outcome after a focal ischemic insult compared to wild-type littermates (Gan et al. 2012).

### Prdx3 and Prdx5

Among the Prdx family, Prdx3 and Prdx5 are distinct in being localized to mitochondria, where they are believed to act as redox sensors and peroxide scavengers. Indeed, they appear to display complementary activities in the matrix with Prdx3 scavenging hydrogen peroxide and Prdx5 more effective against peroxynitrite (De Simoni et al. 2008; Cox et al. 2010). Prdx5, uniquely, can also be associated with peroxisomes, the cytoplasm and the nucleus (Knoops et al. 2011). In the rodent brain, Prdx3 and Prdx5 are constitutively expressed by neurons (Hattori et al. 2003; Jin et al. 2005; Godoy et al. 2011; Goemaere and Knoops 2012). The present results show that in the rat and marmoset retina, Prdx3 and Prdx5 display highly similar patterns of immunoreactivity that are characteristic of mitochondrial markers. Of note, the Prdx3 results in the marmoset reflect those previously reported (Moreira et al. 2008). Prdx3 and Prdx5 were present in RGC axons, with a relatively higher content in the mitochondrial-rich, unmyelinated portion of the nerve. In the rodent brain, Prdx5 displays a wider distribution profile than Prdx3 (Goemaere and Knoops 2012). Our data likewise show that Prdx5 is expressed to a greater degree than Prdx3 in the retina and optic nerve.

#### Implications for disease

In the retina, mitochondrial Prdxs are likely of paramount importance within RGCs. These neurons are hypothesized to contain the greatest number of mitochondria of any CNS neuron, owing to their mitochondrial-rich, unmyelinated axons (Carelli et al. 2013). Mitochondrial dysfunction within RGCs is known to be the primary insult in Leber’s hereditary optic neuropathy (Carelli et al. 2004) and is strongly implicated in the etiology of glaucoma where it might well be both a trigger for, and consequence of, oxidative stress (Chrysostomou et al. 2013; Osborne and del Olmo-Aguado 2013). Given the apparent absence of cytosolic Prdxs in RGCs, the role of mitochondrial Prdxs is likely to be critical in preventing cellular build-up of free radicals. In the brain, expression of Prdx3 increased following transient cerebral ischemia, while intraventricular administration of Prx3 significantly reduced ischemic damage, lipid peroxidation and the release of cytochrome* c* (Hwang et al. 2010). Prdx3 overexpression also protected hippocampal neurons from excitotoxic injury (Hattori et al. 2003). Future work should investigate whether manipulation of mitochondrial Prdxs, in particular Prdx3, affects RGC viability in disease models. The mitochondrial Prdxs were also, as would be expected, abundantly expressed in photoreceptors inner segments. It will be informative to delineate whether Prdx3 becomes hyperoxidised in conditions involving photoreceptor stress.

### Prdx4

Of the Prdxs, Prdx4 has arguably proven the most elusive in terms of definitive establishment of tissue distribution in the brain. Firstly, global expression of Prdx4 is reported to be low (Dammeyer and Arner 2011; Godoy et al. 2011; Kim et al. 2012). Secondly, consensus has not been reached on which cell types express Prdx4. Neurons, oligodendrocytes and astrocytes have all been shown to display positive immunoreactivity (Jin et al. 2005; Rowe et al. 2010; Goemaere and Knoops 2012). Our data show robust, high contrast Prdx4 immunolabeling of macroglial cells of the primate retina and optic nerve, but no discernible association with neurons or oligodendrocytes. These results are supported by unambiguous Western blots of primate and human tissue extracts that attest to the specificity of the antibody. In sections of rat retina and optic nerve, however, no overtly specific Prdx4 immunoreactivity was observed, while Western blots indicated only nominal expression of the protein. There are two possible explanations for the lack of correspondence between rodents and primates: either Prdx4 expression is much lower in young adult rats than aged primates, or, the antibody employed has greater affinity for human Prdx4 than the rat equivalent. In support of the former hypothesis, our qPCR data revealed that Prdx4 mRNA was the least abundant isoform present in rat retina, and lower than all others excepting Prdx3 (which was undetectable by Western blotting) in the optic nerve. Regarding the second hypothesis, Prdx4, like all of the Prdxs, is a highly conserved protein. FASTA protein alignment of the human and rat Prdx4 protein reveals virtually identical sequences. It would seem unlikely that rat Prdx4 fails to recognize an antibody targeted to full length human Prdx4. We did test additional Prdx4 antibodies during the course of this study (see Materials and Methods), but unfortunately they did not provide satisfactory results by Western blotting on rat, marmoset or human tissue extracts. Further investigation of Prdx4 expression in the eye is certainly warranted to confirm or refute the findings presented herein.

### Prdx6

In the retina, Prdx6 was expressed solely by Müller cells and astrocytes. The former cell type, despite spanning the entire thickness of the retina and collectively ensheathing every neuron, comprises only 4 % of the total number of cells in the neuroretina (Jeon et al. 1998), which explains the relative low abundance of Prdx6 mRNA. In the optic nerve, the immunohistochemical findings were consistent with the protein being distributed exclusively to astrocytes. Our results showed a complete correspondence between rat and marmoset with regard to localization of Prdx6 in the retina and optic nerve, and similarly match previously published findings of Prdx6 distribution in human (Power et al. 2002, 2008) rat (Aon-Bertolino et al. 2011) and mouse (Godoy et al. 2011; Goemaere and Knoops 2012) brain, although it should be noted that there is evidence of Prdx6 expression by brain cell types other than astrocytes (Jin et al. 2005; Dammeyer and Arner 2011).

#### Implications for disease

Astrocytes and Müller cells play crucial structural and functional roles in maintenance of barrier function; thus, our finding that these cell types, unique within the retina, express Prdx6 is of obvious relevance to any disease where the blood-retinal barrier is compromised, such as diabetic retinopathy, exudative age-related macular degeneration and arterial and venous occlusions. In the brain, ischemia–reperfusion has been shown to cause increased expression of Prdx6 around hippocampal blood vessels, which was associated with blood–brain barrier breakdown and reactive astrogliosis (Zhang et al. 2013), while upregulated Prx6 has also been documented during Alzheimer’s disease (Power et al. 2008), amyotrophic lateral sclerosis (Strey et al. 2004) and following traumatic injury (Manevich et al. 2014). It will be of interest to learn whether retinal pathologies, particularly those of vasogenic origin, correlate with increased Prdx6 expression.

### Human retina

We have not included a detailed examination of the expression of the Prdx isoforms in human retina and optic nerve for two reasons: firstly, due to a lack of tissue extracts suitable for Western blotting; secondly, due to the fact that the histological preservation of the human eye sections that were available to us for immunohistochemistry was not sufficiently high to engender absolute confidence in any conclusions drawn. With these caveats, it is nevertheless worth stating that our preliminary data (unpublished observations) indicate that the distribution profile in humans closely matches that of the marmoset. Of particular note, Prdx4 was clearly evident in Müller cells and astrocytes, a cellular distribution that correlates with our findings in marmoset retina and human brain.
